# A DNA barcode library for ground beetles of Germany: the genus *Amara* Bonelli, 1810 (Insecta, Coleoptera, Carabidae)

**DOI:** 10.3897/zookeys.759.24129

**Published:** 2018-05-17

**Authors:** Michael J. Raupach, Karsten Hannig, Jérôme Moriniére, Lars Hendrich

**Affiliations:** 1 Institute for Biology and Environmental Sciences, Carl von Ossietzky University Oldenburg, Carl von Ossietzky Straße 9-11, 26111 Oldenburg, Germany; 2 Bismarckstraße 5, 45731 Waltrop, Germany; 3 Taxonomic coordinator – German Barcode of Life (GBOL), Bavarian State Collection of Zoology (SNSB – ZSM), Münchhausenstraße 21, 81247 München, Germany; 4 Sektion Insecta varia, Bavarian State Collection of Zoology (SNSB – ZSM), Münchhausenstraße 21, 81247 München, Germany

**Keywords:** Central Europe, cytochrome *c* oxidase subunit I, German Barcode of Life, mitochondrial DNA, molecular specimen identification, *Zabrus*

## Abstract

The genus *Amara* Bonelli, 1810 is a very speciose and taxonomically difficult genus of the Carabidae. The identification of many of the species is accomplished with considerable difficulty, in particular for females and immature stages. In this study the effectiveness of DNA barcoding, the most popular method for molecular species identification, was examined to discriminate various species of this genus from Central Europe. DNA barcodes from 690 individuals and 47 species were analysed, including sequences from previous studies and more than 350 newly generated DNA barcodes. Our analysis revealed unique BINs for 38 species (81%). Interspecific K2P distances below 2.2% were found for three species pairs and one species trio, including haplotype sharing between *Amara alpina*/*Amara torrida* and *Amara communis/Amara convexior*/*Amara makolskii*. This study represents another step in generating an extensive reference library of DNA barcodes for carabids, highly valuable bioindicators for characterizing disturbances in various habitats.

## Introduction

With the rise of modern sequencing technologies in the early 1990s, DNA sequences have been increasingly used as supplementary markers for species description, identification, and classification (Raupach et al. 2016). In this context, DNA barcoding has become the most popular approach for the assignment of specimens throughout all life stages to described and classified species following the Linnean guidelines (Hebert et al. 2003a, 2003b). In the case of animals, an app. 660 base pair (bp) fragment of the mitochondrial cytochrome *c* oxidase subunit I (COI) gene has been chosen as standardized barcode marker (Hebert et al. 2003a, 2003b). The concept of DNA barcoding is based on a simple assumption: every species will most likely have unique DNA barcodes with low intraspecific variation and interspecific variation exeeds the variability within species, generating a so-called DNA barcoding gap that highly depends on the studied taxonomic groups (Hebert et al. 2003a, 2003b, Čandek and Kuntner 2015, Koroiva and Kvist 2017). In spite of the fact that various effects can limit the usefulness of DNA barcodes and mitochondrial DNA in general, e.g., the presence of pseudogenes or numts (e.g., Bensasson et al. 2001, Leite 2012, Jordal and Kambestad 2014, Haran et al. 2015), heteroplasmy (e.g., Magnacca and Brown 2010, Robinson et al. 2015), effects of *Wolbachia* infections within terrestrial arthropods (e.g., Hurst and Jiggins 2005, Werren et al. 2008, Smith et al. 2012), or general critics on the concept (e.g., Will and Rubinoff 2004, Collins and Cruickshank 2013), numerous studies have demonstrated that DNA barcoding yields excellent results across a broad range of various animal taxa (e.g., Costa et al. 2007, Aliabadian et al. 2013, Knebelsberger et al. 2014, Lobo et al. 2015, Raupach et al. 2015, Barco et al. 2016). Today, barcode data can be easily managed and analysed using the public Barcode of Life data base (BOLD; www.boldsystems.org; Ratnasingham and Hebert 2007). This core data retrieval interface offers various analytical tools, including the Barcode Index Number (BIN) system (Ratnasingham and Hebert 2013).

In term of arthropods, most DNA barcoding studies focus on insects (Raupach and Radulovici 2015), e.g., the Ephemeroptera, Plecoptera and/or Trichoptera (Zhou et al. 2009, Zhou et al. 2011, Ruiter et al. 2013, Moriniére et al. 2017), Heteroptera (Jung et al. 2011, Park et al. 2011, Raupach et al. 2014), Hymenoptera (Smith and Fisher 2009, Smith et al. 2013, Schmidt et al. 2015), Lepidoptera (e.g., Hajibabaei et al. 2006, Hausmann et al. 2011, Hausmann et al. 2013, Kekkonen et al. 2015), and others (e.g., Glover et al. 2010, Moriniére et al. 2014, Hawlitschek et al. 2017). In comparison to the high number of described species, however, the number of studies analysing the Coleoptera (e.g., Greenstone et al. 2011, Woodcock et al. 2013, Pentinsaari et al. 2014, Hendrich et al. 2015, Oba et al. 2015, Rougerie et al. 2015, Han et al. 2016), and in particular the Carabidae or ground beetles (Greenstone et al. 2005, Raupach et al. 2010, 2011, 2016b), is still low.

**Figure 1.**
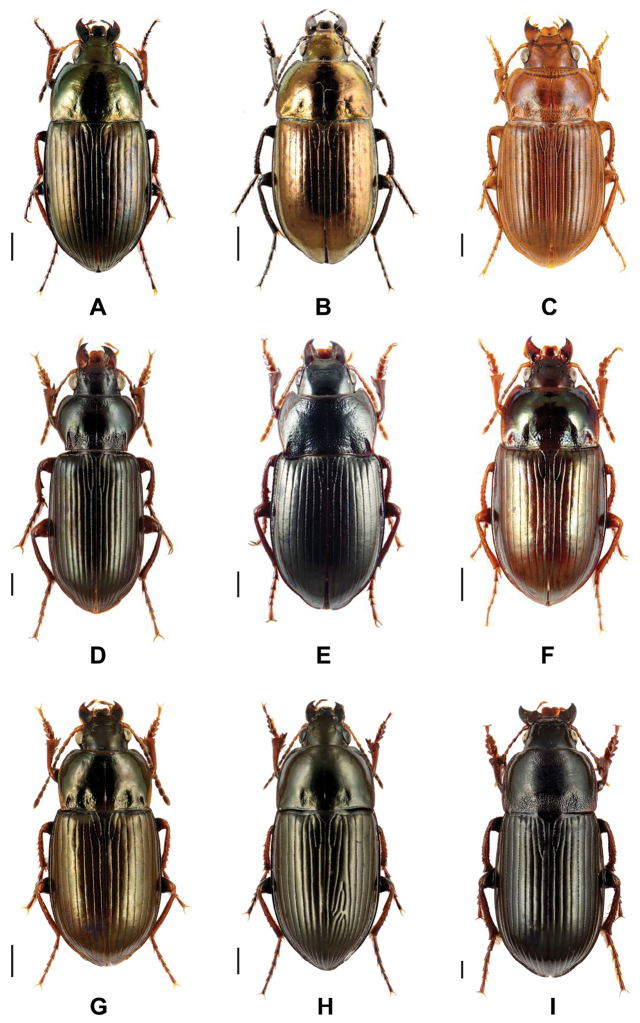
An image collection of some representative species of the analysed ground beetles. **A** Amara (Amara) similata (Gyllenhal, 1810) **B** Amara (Amarocelia) erratica (Duftschmid, 1812) **C** Amara (Bradytus) fulva (Müller, 1776) **D** Amara (Curtonotus) convexiuscula (Marsham, 1802) **E** Amara (Leirides) spectabilis Schau, 1858 **F** Amara (Paracelia) quenseli (Schönherr, 1806) **G** Amara (Xenocelia) cursitans Zimmermann, 1931 **H** Amara (Zezea) kulti Fassati, 1947, and **I** *Zabrus tenebrioides* Goeze, 1777. Scale bars 1 mm. All images were obtained from www.eurocarabidae.de.

Ground beetles represent highly valuable and frequently used bioindicators for the characterization of disturbances in various habitats such as forests, meadows, fens, or river banks (e.g., Lövei and Sunderland 1996, Rainio and Niemelä 2003, Koivula 2011, Kotze et al. 2011). Within the Carabidae, *Amara* Bonelli, 1810 is a large genus in the tribe Zabrini Bonelli, 1810. Many species are Holarctic, but a few are Neotropical or occur in Eastern Asia. About 150 European species are known (Luff 2007), with 52 recorded for Germany (Trautner et al. 2014). Beetles of this genus are typically characterized by their rather oval and parallel-sided form, with females that are often somewhat duller than the males and may even differ in body shape (Luff 2007) (Fig. 1). While ground beetles are mostly carnivorous, numerous *Amara* species feed on plant seeds as both larvae and adults (e.g., Hůrka 1996, Jørgensen and Toft 1997, Holland 2002, Klimeš and Saska 2010), although some species consume seeds only as a supplement to their predominantly predatory diet (e.g., Goldschmidt and Toft 1997, Holland 2002, Koprdova et al. 2008). They typically require dry habitats, uncultivated areas and open vegetation on light soils, such as sand, gravel, or chalk (e.g., Kromp 1989, Thomas et al. 2001). As a consequence of their more or less homogenous habitus and very subtle morphological differences between species (e.g., the shape of the pronotum or coloration of antennomeres), *Amara* is known as the most challenging genus of ground beetles in terms of species identification in Central Europe. Nevertheless, Fritz Hieke (1930–2015) devoted his scientific career to this genus and thoroughly cleared up the difficult taxonomic assessment of this genus at all levels (e.g., Hieke 1984, 1988, 2005). In this context he published a list of valid names and their synonyms, with over 560 specific and subspecific, and 47 subgeneric names (Hieke 1995, 2011).

Here we present the next step in building-up a comprehensive DNA barcode library of Central European species of ground beetles as part of the German Barcode of Life project (GBOL), focusing on the genus *Amara*. The analysed barcode library included 46 *Amara* species as well as one species of *Zabrus* Clairville, 1806 which represents the second genus of the tribe Zabrini known from Central Europe. Four species (*Amara littorea* Thomson, 1857, *Amara makolskii* Roubal, 1923, *Amara sabulosa* Audinet-Serville, 1821, and *Amara spectabilis* Schaum, 1858) were not covered by previous studies (Raupach et al. 2010, Pentinsaari et al. 2014, Hendrich et al. 2015). In summary, 358 new barcodes were generated and a total number of 690 DNA barcodes examined.

## Material and methods

### Sampling of specimens

All new studied beetles were collected between 1997 and 2017 using various sampling methods (e.g., hand collecting, pitfall traps). Beetles were stored in ethanol (96%) and determined by two of the authors (KH, MJR), K.-H. Kielhorn (Berlin, Germany) and F. Köhler (Bonn, Germany) using the keys in Hieke (2006) or Paill (2016). In total, 358 new DNA barcodes of 37 species were generated. Furthermore, 332 DNA barcodes of three previous studies (Raupach et al. 2010: 17 specimens, 5 species; Pentinsaari et al. 2014: 113 specimens, 34 species; Hendrich et al. 2015: 202 specimens, 32 species) were included, generating a data set of 690 DNA barcodes from 47 species in total. Five of the studied species are not known from Germany, including *Amara alpina* (Paykull, 1790) (*n* = 3; collected in Finland, see Pentinsaari et al. 2014), *Amara hyperborea* Dejean, 1831 (*n* = 1; collected in Finland, see Pentinsaari et al. 2014), *Amara interstitialis* Dejean, 1828 (*n* = 1; collected in Finland, see Pentinsaari et al. 2014), *Amara spectabilis* Schaum, 1858 (*n* = 3, collected in Austria), and *Amara torrida* Panzer, 1796 (*n* = 4; collected in Finland, see Pentinsaari et al. 2014). The number of specimens per species ranged from one (6 species) to a maximum of 55 for *Amara aenea* (De Geer, 1774). Most beetles were collected in Germany (*n* = 513, 74.4%), whereas various specimens from other countries were included for comparison: Finland (99, 14.4%), Austria (41, 5.9%), Italy (12, 1.7%), Sweden (7, 1%), Estonia (4, 0.6%), France (4, 0.6%), Czech Republic (3, 0.4%), Denmark (3, 0.4%), Belgium (2, 0.3%), and Slovenia (2, 0.3%).

### DNA barcode amplification, sequencing, and data depository

All laboratory operations were carried out, following standardized protocols for COI amplification and sequencing (Ivanova et al. 2006, deWaard et al. 2008) at the Canadian Center for DNA Barcoding (CCDB), University of Guelph, the molecular labs of the Zoologisches Forschungsmuseum Alexander Koenig in Bonn, the German Centre of Marine Biodiversity Research, Senckenberg am Meer, in Wilhelmshaven, or the working group Systematics and Evolutionary Biology at the Carl von Ossietzky University Oldenburg, all in Germany. Photos were taken from each studied beetle before molecular work was performed. One or two legs of one body side were removed for the subsequent DNA extraction which was performed using the QIAmp Tissue Kit (Qiagen GmbH, Hilden, Germany) or NucleoSpin® Tissue Kit (Macherey-Nagel, Düren, Germany), following the extraction protocol.

Detailed information about primers used, PCR amplification and sequencing protocols is given in a previous publication (see Raupach et al. 2016b). All purified PCR products were cycle-sequenced and sequenced in both directions at contract sequencing facilities (Macrogen, Seoul, Korea, or GATC, Konstanz, Germany), using the same primers as used in PCR. Double stranded sequences were assembled and checked for mitochondrial pseudogenes (numts) analysing the presence of stop codons, frameshifts as well as double peaks in chromatograms with the Geneious version 7.0.4 program package (Biomatters, Auckland, New Zealand) (Kearse et al. 2012). For verification, BLAST searches (nBLAST, search set: others, program selection: megablast) were performed to confirm the identity of all new sequences as ground beetle sequences based on already published sequences (high identity values, very low E-values) (Zhang et al. 2000, Morgulis et al. 2008).

Comprehensive voucher information, taxonomic classifications, photos, DNA barcode sequences, primer pairs used and trace files (including their quality) are publicly accessible through the public data set “DS-BAAMA” (Dataset ID: dx.doi.org/10.5883/DS-BAAMA) on the Barcode of Life Data Systems ( BOLD; www.boldsystems.org) (Ratnasingham and Hebert 2007). Finally, all new barcode data were deposited in GenBank (accession numbers: MH300683–MH300903).

### DNA barcode analysis

The analysis tools of the BOLD workbench were employed to calculate the nucleotide composition of the sequences and distributions of Kimura-2-parameter distances (K2P; Kimura 1980) within and between species (align sequences: BOLD aligner; ambiguous base/gap handling: pairwise deletion). All barcode sequences became subject of the Barcode Index Number (BIN) system implemented in BOLD which clusters DNA barcodes in order to produce operational taxonomic units that closely correspond to species (Ratnasingham and Hebert 2013). A threshold of 2.2% was applied for a rough differentiation between intraspecific and interspecific distances based on Ratnasingham and Hebert (2013). It should be noted that the BIN assignments on BOLD are constantly updated as new sequences are added. Therefore, individual BINs can be split or merged in light of new data (Ratnasingham and Hebert 2013).

Furthermore, all sequences were aligned using MUSCLE (Edgar 2004) and analysed using a neighbour-joining cluster analysis (NJ; Saitou and Nei 1987) based on K2P distances with MEGA7.0.21 (Kumar et al. 2016). Non-parametric bootstrap support values were obtained by resampling and analying 1,000 replicates (Felsenstein 1985). It should be explicitly noted that this analysis is not intended to be phylogenetic. Instead of this, the shown topology represents a graphical visualization of DNA barcode divergences and putative species cluster. Finally, statistical maximum parsimony networks were constructed for species pairs with interspecific distances <2.2% with TCS 1.21 based on default settings (Clement et al. 2000) as part of the software package of PopART v.1.7 (Leigh and Bryant 2015). Such networks allow the identification of haplotype sharing between species as a consequence of recent speciation or on-going hybridization processes (e.g., Raupach et al. 2010).

## Results

In total, 690 DNA barcode sequences of 47 carabid beetle species were examined. A full list of the species is presented in the supporting information (Suppl. material 1). In total, 46 species of the genus *Amara* were studied, with 41 (79%) of the 52 species documented for Germany. Five analysed species, *Amara alpina* (Paykull, 1790) (*n* = 3), *Amara hyperborea* Dejean, 1831 (*n* = 1), *Amara interstitialis* Dejean, 1828 (*n* = 1), *Amara spectabilis* Schaum, 1858 (*n* = 3), and *Amara torrida* Panzer, 1796 (*n* = 4), are not known from Germany. All these specimens were collected from other countries (see above). Fragment lengths ranged from 307 (*n* =14) to a full length of 657 bp. Base frequencies analysis revealed low GC-contents (average: 32%) for the barcode fragment, as it is known from insects and other arthropods. The individual mean nucleotide contents were A = 0.29, C = 0.15, G = 0.17, and T = 0.39. Intraspecific K2P distances ranged from zero to 2.18% (*Amara bifrons* (Gyllenhal, 1810)). Interspecific K2P distances had values between zero and a maximum of 10.06%.

The BIN analyses were performed on January 11^th^ 2018. Unique BINs were revealed for 38 species (81%). Three species pairs shared a BIN: *Amara alpina* Paykull, 1790 and *Amara torrida* (Panzer, 1796) were both included in ACF5385, *Amara familiaris* (Duftschmid, 1812) and *Amara lucida* (Duftschmid, 1812) in AAC4901, and *Amara ovata* (Fabricius, 1792) and *Amara similata* (Gyllenhal, 1820) in AAJ5377. Furthermore, one BIN (ACF1000) contained three species: *Amara communis* (Panzer, 1797), *Amara convexior* Stephens, 1828, and *Amara makolskii* Roubal, 1923 (the so-called *Amara communis* complex).Interspecific distances of zero were found for *Amara alpina* and *Amara torrida* as well as for *Amara communis*, *Amara convexior* and *Amara makolskii*.

The NJ analyses based on K2P distances revealed non-overlapping clusters with bootstrap support values >90% for 33 species (70% of all studied species) with more than one studied specimen (Fig. 2). A comprehensive topology is presented in the supporting information (Suppl. material 2).

**Figure 2.**
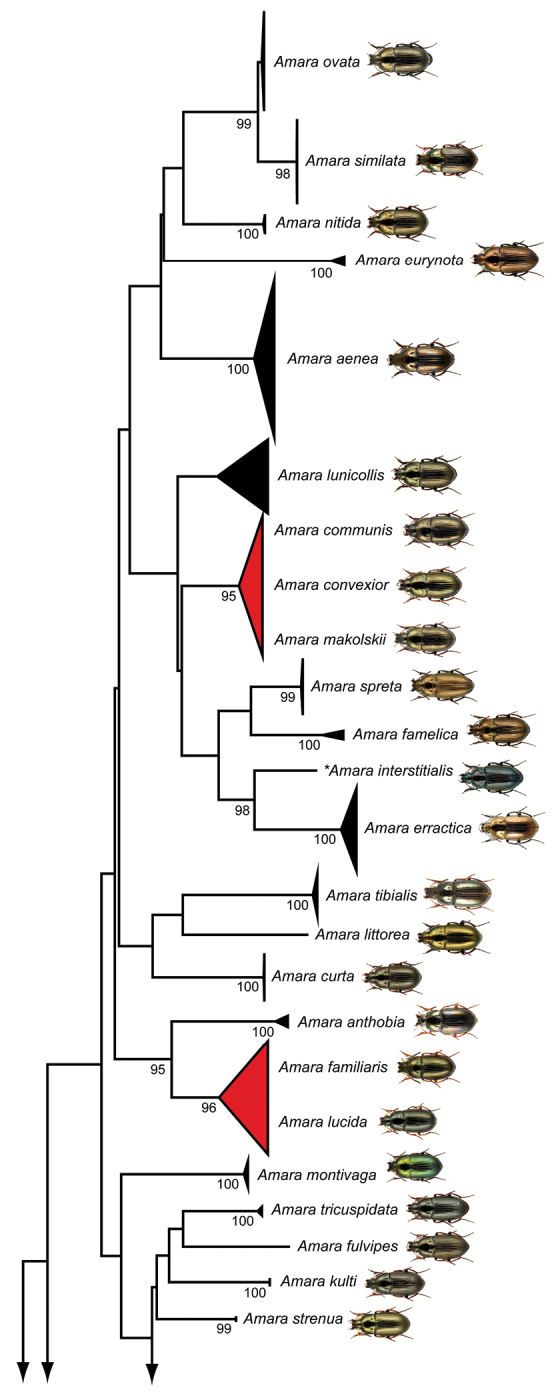
Neighbor joining topology of the analysed ground beetle species based on Kimura 2-parameter distances. Triangles show the relative number of individual’s sampled (height) and sequence divergence (width). Red triangles indicate species pairs with interspecific distances <2.2%. Numbers next to nodes represent non-parametric bootstrap values >90% (1,000 replicates). Asterisks indicate species not recorded in Germany. All images were obtained from www.eurocarabidae.de.

**Figure 2.**
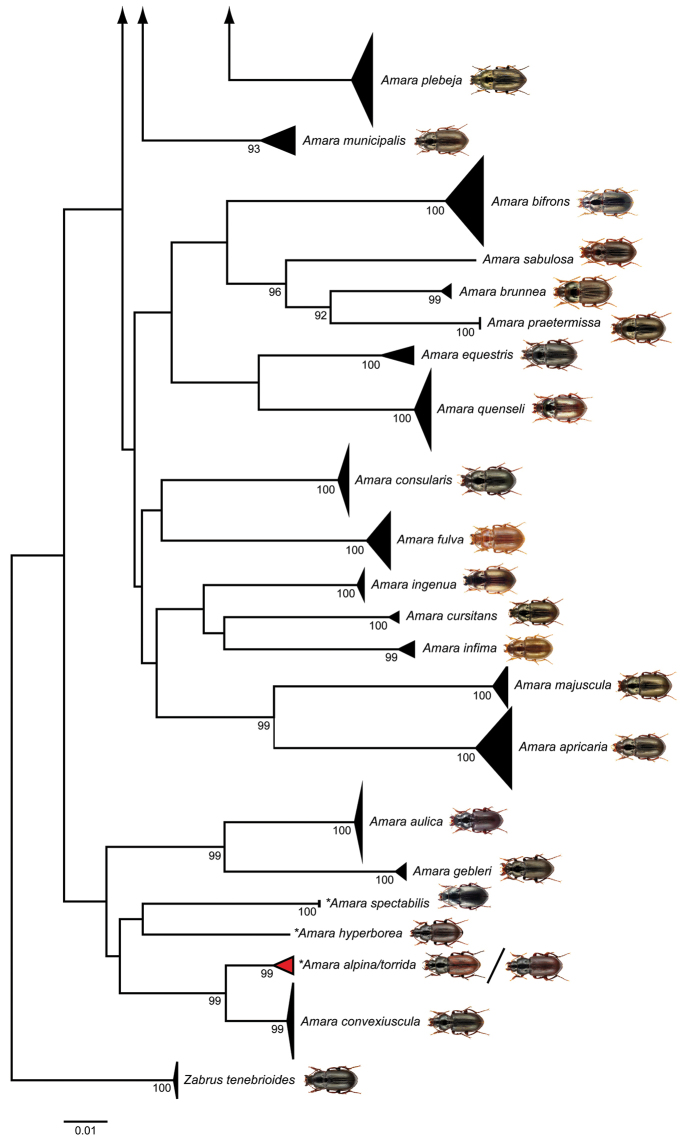
Continue.

**Figure 3.**
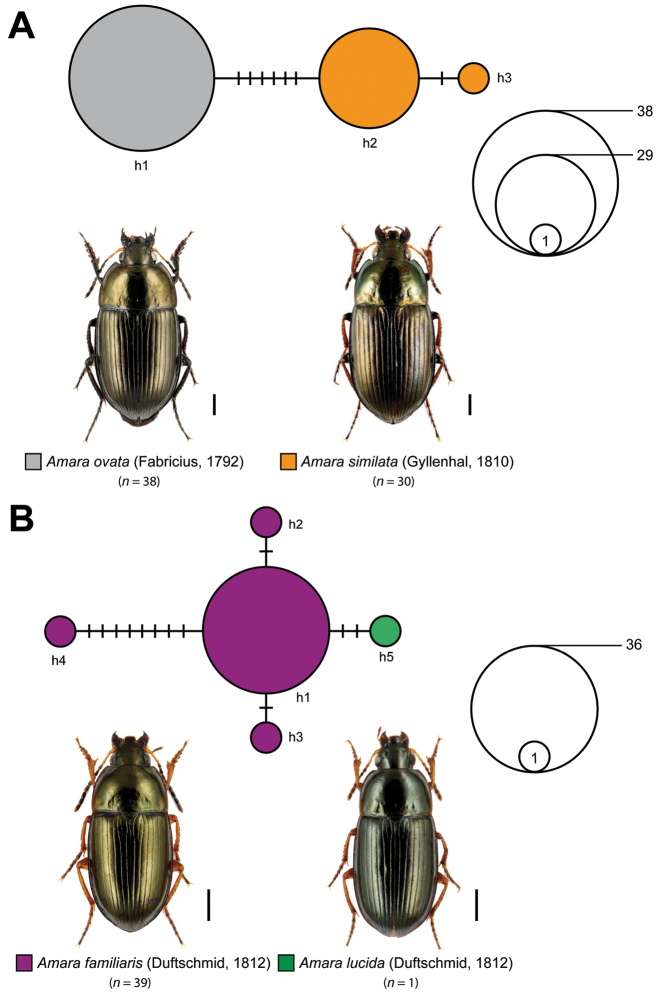
Maximum statistical parsimony networks of two species pairs: **A** *Amara ovata* (Fabricius, 1792) and *Amara similata* (Gyllenhal, 1810), and **B** *Amara familiaris* (Duftschmid, 1812) and *Amara lucida* (Duftschmid, 1812). Used parameters included default settings for connection steps whereas gaps were treated as fifth state. Each line represents a single mutational change whereas small black lines indicate missing haplotypes. The numbers of analysed specimens (*n*) are listed, the diameter of the circles is proportional to the number of haplotypes sampled (see given open half circles with numbers). Scale bars 1 mm. Beetle images were obtained from www.eurocarabidae.de.

**Figure 4.**
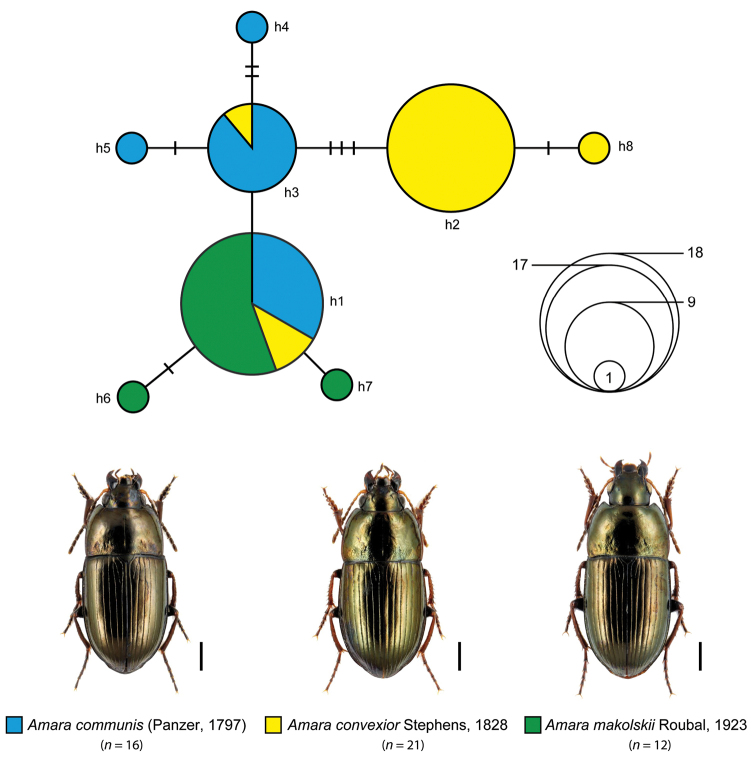
Maximum statistical parsimony network of the *Amara communis* complex. Used parameters included default settings for connection steps whereas gaps were treated as fifth state. Each line represents a single mutational change whereas small black lines indicate missing haplotypes. The numbers of analysed specimens (*n*) are listed, the diameter of the circles is proportional to the number of haplotypes sampled (see given open half circles with numbers). Scale bars 1 mm. Beetle images were obtained from www.eurocarabidae.de.

**Figure 5.**
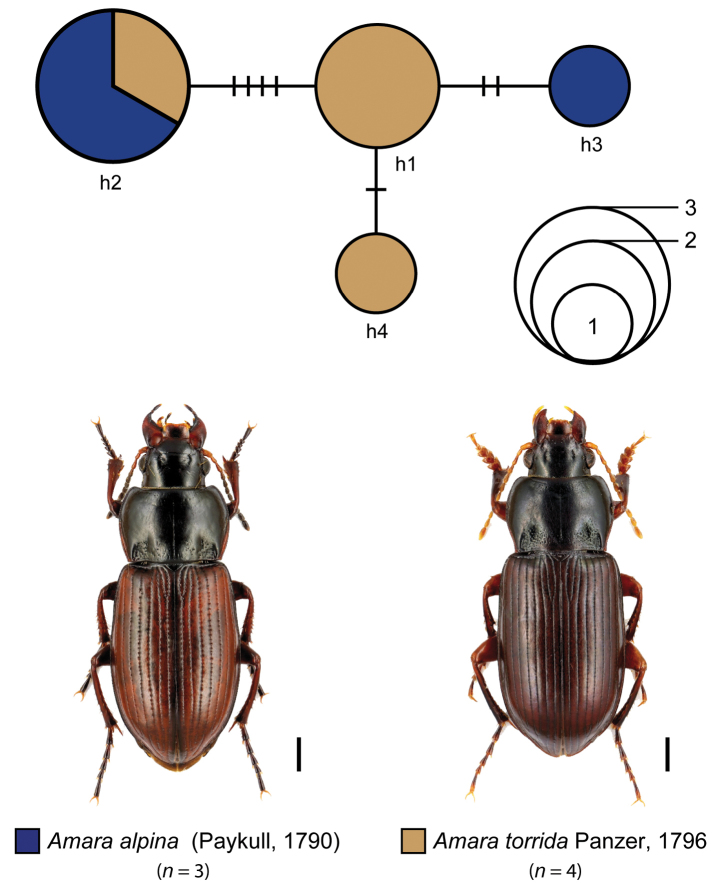
Maximum statistical parsimony network of *Amara alpina* (Paykull, 1790) and *Amara torrida* Panzer, 1796. Used parameters included default settings for connection steps whereas gaps were treated as fifth state. Each line represents a single mutational change whereas small black lines indicate missing haplotypes. The numbers of analysed specimens (*n*) are listed, the diameter of the circles is proportional to the number of haplotypes sampled (see given open half circles with numbers). Scale bars 1 mm. Beetle images were obtained from www.eurocarabidae.de.

Our statistical maximum parsimony analysis revealed closely related haplotypes for *Amara ovata* (Fabricus, 1792) and *Amara similata* (Gyllenhal, 1810) (Fig. 3a). The dominant haplotypes of both species (*Amara ovata*: h1, *Amara similata*: h2) were separated by six mutational steps. An even lower number of mutational steps were found between *Amara familiaris* (Duftschmid, 1812) and *Amara lucida* (Duftschmid, 1812) (Fig. 3b): the only examined specimen of *Amara lucida* (h5) was separated from the dominant haplotype of *Amara familiaris* (h1) by two mutational steps. Furthermore, multiple haplotypes shared by more than one species were found in the *Amara communis* complex (*n* = 49; Fig. 4) and for *Amara alpina* (*n* = 3) with *Amara torrida* (*n* = 4) (Fig. 5). For the *Amara communis* complex, eight different haplotypes with two dominant ones (h1, h2) were identified. Whereas haplotype h1 was shared by 18 specimens with all three species (*Amara communis*: *n* = 6, *Amara convexior*: *n* = 2, *Amara makolskii*: *n* = 10), haplotype h2 was found exclusively in specimens of *Amara convexior* (*n* = 17). Haplotype h3, located between h1 and h2 in the network, was shared by specimens of *Amara communis* (*n* = 8) and *Amara convexior* (*n* = 1). In addition, five haplotypes represented by one specimen only (singletons) were located at the periphery of the network (*Amara communis*: h4, h5, *Amara convexior*: h8, *Amara makolskii*: h6, h7). In the case of *Amara alpina* and *Amara torrida*, the statistical maximum parsimony analysis revealed four haplotypes, with one haplotype (h2) shared by specimens of both species (*Amara alpina*: 2 specimens, *Amara torrida*: 1 specimen). This haplotype was separated by four additional steps from haplotype h1 that was restricted to specimens of *Amara torrida*. Furthermore, two singletons (h3: two additional mutational steps; h4: one additional mutational step) were connected with haplotype h1, generating a compact network that contained only a few mutational steps.

## Discussion

Within the past few years, DNA-based approaches have become more and more popular for the assessment of biodiversity and identification of specimens, in particular where the traditional morphology-based identification has proved problematic (Taberlet et al. 2012). As a consequence of this development and the rise of new concepts (Hebert et al. 2003a, 2003b), the analysis of single specimens, bulk samples (metabarcoding) or environmental DNA (eDNA) will be performed routinely as part of modern species diversity assessment studies in the near future (e.g., Scheffers et al. 2012, Cristescu 2014, Kress et al. 2015). However, such studies highly rely on comprehensive on-line sequence libraries that act as references (e.g., Brandon-Mong et al. 2015, Creer et al. 2016, Staats et al. 2016). Therefore, our DNA barcode library represents an important step for the molecular characterization of ground beetles in Central Europe and adjacent regions. The current results demonstrate that DNA barcodes distinguish Central European species of the taxonomically challenging genus *Amara* remarkably well. Our analysis revealed unique BINs for 38 (81%) of the 47 analysed species. The results coincide with high rates of successful species identification of previous barcoding studies on ground beetles (Raupach et al. 2010, 2011, Pentinsaari et al. 2014, Hendrich et al. 2015, Raupach et al. 2016b). In contrast to other carabid genera, e.g., *Bembidion* Latreille, 1802 (Raupach et al. 2016b) or *Calathus* Bonelli, 1810 (Hendrich et al. 2015), no evidence was found for high intraspecific distances (above 2.2%) within the analysed *Amara* species. In contrast to this, low intraspecific distances (below 2.2%) and shared haplotypes for various species pairs were revealed. Such low distances are typically indicative of a recent ancestry and/or ongoing gene flow for various species pairs (e.g., Tautz et al. 2003, Frezal and Leblois 2008, Raupach et al. 2010). We will discuss these cases in more detail.

### I. *Amara ovata* (Fabricius, 1792) and *Amara similata* (Gyllenhal, 1810)

Both species are abundant and widespread members of the subgenus Amara, with a trans-Palearctic distribution from Europe to Eastern Siberia (e.g., Lindroth 1986, Hůrka 1996, Hieke 2006). Using morphological traits, both species are best separated on the shape of the pronotum (Hieke 1975, Lindroth 1986, Hieke 2006). Nevertheless, a close relationship of both species has been already suggested in the past (e.g., Lindroth 1986, Luff 2007). Our analysis clearly supports this view. In spite of the fact that both species have the same BIN, they form distinct clusters separated by six mutational steps (Fig. 3A). Consequently, all examined specimens can be assigned to both species without doubt. However, it should be noted that the amount of intraspecific variation of DNA barcode sequences (and mitochondrial DNA in general) can correlate with the geographical scale of sampling (e.g., Wiemers and Fiedler 2007, Bergsten et al. 2012 but see Huemer et al. 2014). For this study, all studied specimens were sampled in Europe (*Amara ovata*: 1 specimen from Belgium, 1 from Italy, 6 from Finland, 30 from Germany; *Amara similata*: 3 specimens from Finland, 27 from Germany). Only the analysis of additional beetles from other regions, e.g., Central and Eastern Asia, will show if both species can be identified across their complete distribution ranges without doubt.

### II. *Amara familiaris* (Duftschmid, 1812) and *Amara lucida* (Duftschmid, 1812)

Similar to the previous species, *Amara familiaris* and *Amara lucida* are widespread species of the subgenus Amara with a Palearctic (*Amara familiaris*) or West Palearctic (*Amara lucida*) distribution (Hůrka 1996, Hieke 2006). From a morphological perspective, both species are very similar, being black with a greenish or brassy metallic reflection (e.g., Luff 2007). However, specimens of *Amara lucida* are somewhat smaller and a little narrower than beetles of *Amara familiaris*, but the only useful morphological traits for species identification are differences within the front angles of the pronotum (e.g., Lindroth 1986, Hůrka 1996, Hieke 2006). Not surprisingly, the given DNA barcode data confirm the supposed closed relationship (Fig. 3B), but unfortunately only one specimen of *Amara lucida* has been examined so far. More beetles of this species should be studied in detail in the near future in order to validate if two distinct clusters exist or haplotype sharing occurs.

### III. The *Amara communis* complex

Within the genus *Amara*, the *Amara communis* complex represents one of the most challenging and controversial group of species in Europe. The complex consists of four very similar and closely related species of the subgenus Amara: *Amara communis* (Panzer, 1797), *Amara convexior* Stephens, 1828, *Amara makolskii* Roubal, 1923, and *Amara pulpani* Kult, 1949. All species are characterized by the combination of various morphological traits including the presence of a scutellar stria, deepened and apically widened elytral striae, and the coloration of antennomere 2 and 3 (Hůrka 1996, Hůrka and Rúžičkova 1999, Paill 2016). The specific status of *Amara communis* and *Amara convexior* has been acknowledged for a long time (e.g., Hieke 2006). Both are, similar to other species of this genus, widespread and abundant species with a Palearctic (*Amara communis*) or West Palearctic (*Amara lucida*) distribution (Hůrka 1996, Hieke 2006). In contrast to this, *Amara makolsii* und *Amara pulpani* were considered as synonyms of *Amara communis* (e.g., Lindroth 1986, Hieke 2006, but see Gersdorf and Kuntze 1957, Burakowski 1967). Nevertheless, both species were accepted as valid species some years ago (Hůrka 1996, Löbl and Smetana 2017), but their distribution is still insufficiently documented (e.g., Hůrka 1996, Paill 2003, Schmidt 2004, Schäfer 2005, Gebert 2009, Müller-Kroehling 2013, Trautner et al. 2014). Not surprisingly, the DNA barcode data revealed multiple haplotype sharing between all three studied species, preventing correct species identification (Fig. 4). Unfortunately, DNA barcodes of *Amara pulpani* are currently missing and have to be generated in the future. Nevertheless, we strongly recommend a comprehensive analysis of fast evolving nuclear markers, e.g., microsatellites or SNPs, from specimens of all four species from different localities in order to evaluate if already distinct species exist or hybridization events still take place.

### IV. *Amara alpina* Paykull, 1790 and *Amara torrida* (Panzer, 1796)

All data of both species were part of a previous study (Pentinsaari et al. 2014), but not discussed in detail. The two species are part of the subgenus Curtonotus, show a widespread circumpolar distribution, and are suggested as closely related (Lindroth 1986). In general, specimens of *Amara alpina* can be separated from *Amara torrida* by the color of the appendages and the pronotal form (Lindroth 1986). Similar to the *Amara communis* complex (see above), haplotype sharing prevents a valid discrimination of both species by the means of DNA barcoding (Fig. 5). Again, more specimens and other, especially nuclear markers, have to be studied to analyse if *Amara alpina* and *Amara torrida* still hybridize or distinct species exist.

## Conclusions

Used alone or in combination with DNA metabarcoding on environmental samples (Taberlet et al. 2012), DNA barcoding is becoming a standard for basic and applied research in ecology, evolution and conservation across taxa, communities and ecosystems (Zinger and Philippe 2016). In this context, our study clearly encourages the use of DNA barcodes for the identification of ground beetles species of the taxonomically difficult genus *Amara*. However, DNA barcodes of additional eleven *Amara* species documented for Germany are currently missing. The analysis of these missing species may include other, so far undetected problematic cases. For example, *Amara chaudoiri* Schaum, 1858 and *Amara concinna* Zimmermann, 1832 are morphologically very similar species. Nevertheless, our data set and results represent another important step in building-up a comprehensive barcode library for the Carabidae in Germany and Central Europe which can be used in modern molecular biodiversity assessment studies. Despite the fact that DNA barcoding failed to deliver a valid species identification for some species in this study, it narrows the options to a pair (or in one case trio) of closely related species. Especially for the almost impossible identification of immature stages and/or females within various species of *Amara*, this is a very encouraging result.

## Supplementary materials

Supplementary material 1 Barcode analysis using the BOLD workbench Data type: Data table. Explanation note: Molecular distances based on the Kimura 2-parameter model of the analysed specimens of the studied species of the genera *Amara* and *Zabrus*. Divergence values were calculated for all studied sequences, using the Nearest Neighbour Summary implemented in the Barcode Gap Analysis tool provided by the Barcode of Life Data System (BOLD). Align sequencing option: BOLD aligner (amino acid based HMM), ambiguous base/gap handling: pairwise deletion. ISD = intraspecific distance. BINs are based on the barcode analysis from 15-01-2018. Asterisks indicate species not recorded from Germany. Species pairs with interspecific distances <2.2% are marked in bold. This is an open access article distributed under the terms of the Creative Commons Attribution License (CC BY 4.0), which permits unrestricted use, distribution, and reproduction in any medium, provided the original author and source are credited.Michael J. Raupach, Karsten Hannig, Jérôme Moriniére, Lars Hendrich

Supplementary material 2 Neighbour joining topology Data type: Neighbour joining topology. Explanation note: Neighbour joining phylogram of all analysed ground beetle specimen based on Kimura 2-parameter distances. Individuals are classified using ID numbers from BOLD and species name. Numbers next to nodes represent non-parametric bootstrap values (1,000 replicates, in %). This is an open access article distributed under the terms of the Creative Commons Attribution License (CC BY 4.0), which permits unrestricted use, distribution, and reproduction in any medium, provided the original author and source are credited.Michael J. Raupach, Karsten Hannig, Jérôme Moriniére, Lars Hendrich
