# Supplementary material for: A DNA barcode library for ground beetles of Germany: the genus Amara Bonelli, 1810 (Insecta, Coleoptera, Carabidae)
Source: Zookeys. 2018 May 17;(759):57–80. doi: 10.3897/zookeys.759.24129 (PMC5968077; doi:10.3897/zookeys.759.24129)
Supplement: Supplementary material 1 — Barcode analysis using the BOLD workbench [file zookeys-759-057-s001.docx]

| No. | Species | *n* | BIN | Max. intraspecific pairwise  K2P distance (%) | Min. interspecific pairwise  K2P distance (%) | Nearest Species |
| --- | --- | --- | --- | --- | --- | --- |
| 1 | *Amara (Amara) aenea* (Degeer, 1774) | 55 | AAC4902 | 1.17 | 4.15 | *Amara ovata* |
|  | ****Amara (Curtonotus) alpina* Paykull, 1790** | **3** | **ACF5385** | **1.08** | **0** | ***Amara torrida*** |
|  | *Amara (Amara) anthobia* A. et G.B. Villa, 1833 | 4 | ACC9688 | 0.49 | 3.51 | *Amara familiaris* |
|  | *Amara (Bradytus) apricaria* (Paykull, 1790) | 32 | AAH2780 | 2.18 | 9.67 | *Amara majuscula* |
| 5 | *Amara (Curtonotus) aulica* (Panzer, 1797) | 26 | AAP8857 | 0.49 | 7.08 | *Amara gebleri* |
|  | *Amara (Celia) bifrons* (Gyllenhal, 1810) | 35 | AAO4836 | 2.18 | 9.01 | *Amara praetermissa* |
|  | *Amara (Acrodon) brunnea* (Gyllenhal, 1810) | 5 | ACE2131 | 0.34 | 5.3 | *Amara praetermissa* |
|  | ***Amara (Amara) communis* (Panzer, 1797)** | **16** | **ACF1000** | **0.64** | **0** | ***Amara makolskii*** |
|  | *Amara (Bradytus) consularis* (Duftschmid, 1812) | 25 | AAO0086 | 0.49 | 6.46 | *Amara municipalis* |
| 10 | ***Amara (Amara) convexior* Stephens, 1828** | **21** | **ACF1000** | **0.93** | **0** | ***Amara makolskii*** |
|  | *Amara (Curtonotus) convexiuscula* (Marsham, 1802) | 31 | AAP9134 | 0.32 | 2.9 | *Amara torrida* |
|  | *Amara (Xenocelia) cursitans* Zimmermann, 1931 | 4 | ACF5818 | 0.62 | 6.13 | *Amara municipalis* |
|  | *Amara (Amara) curta* Dejean, 1828 | 17 | AAP5966 | 0 | 5.08 | *Amara littorea* |
|  | *Amara (Percosia) equestris* (Duftschmid, 1812) | 7 | ACC3111 | 1.71 | 7.27 | *Amara quenseli* |
| 15 | *Amara (Amarocelia) erratica* (Duftschmid, 1812) | 29 | ACE3664 | 1.08 | 2.97 | *Amara interstitialis* |
|  | *Amara (Amara) eurynota* (Panzer, 1797) | 3 | AAY7576 | 0.31 | 4.81 | *Amara nitida* |
|  | *Amara (Amara) famelica* Zimmermann, 1832 | 3 | ACE0697 | 0.77 | 2.48 | *Amara spreta* |
|  | ***Amara (Amara) familiaris* (Duftschmid, 1812)** | **39** | **AAC4901** | **1.92** | **0.15** | ***Amara lucida*** |
|  | *Amara (Bradytus) fulva* (O. F. Müller, 1776) | 22 | AAP7213 | 1.27 | 8.31 | *Amara municipalis* |
| 20 | *Amara (Zezea) fulvipes* (Audinet-Serville, 1821) | 1 | AAO4838 | 0 | 3.46 | *Amara tricuspidata* |
|  | *Amara (Curtonotus) gebleri* Dejean, 1831 | 6 | AAM0524 | 0.46 | 7.08 | *Amara aulica* |
|  | **Amara (Curtonotus) hyperborea* Dejean, 1831 | 1 | ACE3468 | 0 | 6.72 | *Amara convexiuscula* |
|  | *Amara (Celia) infima* (Duftschmid, 1812) | 6 | AAQ2242 | 0.46 | 3.8 | *Amara plebeja* |
|  | *Amara (Xenocelia) ingenua* (Duftschmid, 1812) | 11 | ABW5839 | 0.31 | 6.58 | *Amara municipalis* |
| 25 | **Amara (Amarocelia) interstitialis* Dejean, 1828 | 1 | ACO9876 | 0 | 2.97 | *Amara erratica* |
|  | *Amara (Zezea) kulti* Fassati, 1947 | 3 | ACC1918 | 0 | 3.63 | *Amara tricuspidata* |
|  | *Amara (Amara) littorea* Thomson, 1857 | 1 | ACZ0349 | 0 | 5.08 | *Amara tibialis* |
|  | ***Amara (Amara) lucida* (Duftschmid, 1812)** | **1** | **AAC4901** | **0** | **0.15** | ***Amara familiaris*** |
|  | *Amara (Amara) lunicollis* Schiödte, 1837 | 26 | AAO3880 | 0.66 | 2.33 | *Amara makolskii* |
| 30 | *Amara (Bradytus) majuscula* (Chaudoir, 1850) | 16 | AAP9133 | 0.64 | 9.67 | *Amara apricaria* |
|  | ***Amara (Amara) makolskii* Roubal, 1923** | **12** | **ACF1000** | **0.31** | **0** | ***Amara communis*** |
|  | *Amara (Amara) montivaga* Sturm, 1825 | 9 | AAW5994 | 0.15 | 4.9 | *Amara makolskii* |
|  | *Amara (Xenocelia) municipalis* (Duftschmid, 1812) | 11 | AAZ5039 | 0.75 | 1.89 | *Amara plebeja* |
|  | *Amara (Amara) nitida* Sturm, 1825 | 7 | AAW5995 | 0.16 | 3.16 | *Amara ovata* |
| 35 | ***Amara (Amara) ovata* (Fabricius, 1792)** | **38** | **AAJ5377** | **0** | **0.68** | ***Amara similata*** |
|  | *Amara (Zezea) plebeja* (Gyllenhal, 1810) | 35 | AAZ7709 | 0.62 | 1.89 | *Amara municipalis* |
|  | *Amara (Acrodon) praetermissa* (Sahlberg, 1827) | 5 | ACC1917 | 0 | 5.3 | *Amara brunnea* |
|  | *Amara (Paracelia) quenseli (*Schönherr, 1806) | 30 | AAB2273 | 0.93 | 7.27 | *Amara equestris* |
|  | *Amara (Celia) sabulosa* Audinet-Serville, 1821 | 1 | ACZ0348 | 0 | 6.93 | *Amara brunnea* |
| 40 | ***Amara (Amara) similata* (Gyllenhal, 1810)** | **30** | **AAJ5377** | **0.17** | **0.68** | ***Amara ovata*** |
|  | **Amara (Leirides) spectabilis* Schaum, 1858 | 3 | ACR9024 | 0 | 7.81 | *Amara hyperborea* |
|  | *Amara (Amara) spreta* Dejean, 1831 | 20 | ACJ7875 | 0 | 2.48 | *Amara famelica* |
|  | *Amara (Zezea) strenua* Zimmermann, 1832 | 2 | AAY7568 | 0 | 3.71 | *Amara kulti* |
|  | *Amara (Amara) tibialis* (Paykull, 1798) | 15 | ABX3825 | 0 | 5.08 | *Amara littorea* |
| 45 | ****Amara (Curtonotus) torrida* (Panzer, 1796)** | **4** | **ACF5385** | **0.92** | **0** | ***Amara alpina*** |
|  | *Amara (Zezea) tricuspidata* Dejean, 1831 | 3 | AAO4863 | 0 | 3.46 | *Amara fulvipes* |
|  |  |  |  |  |  |  |
| 47 | *Zabrus tenebrioides* Goeze, 1777 | 15 | ACB9072 | 0.31 | 10.06 | *Amara torrida* |
