# Supplementary material for: A DNA barcode library for ground beetles of Germany: the genus Amara Bonelli, 1810 (Insecta, Coleoptera, Carabidae)
Source: Zookeys. 2018 May 17;(759):57–80. doi: 10.3897/zookeys.759.24129 (PMC5968077; doi:10.3897/zookeys.759.24129)
Supplement: Supplementary material 2 — Neighbour joining topology [file zookeys-759-057-s002.pdf]

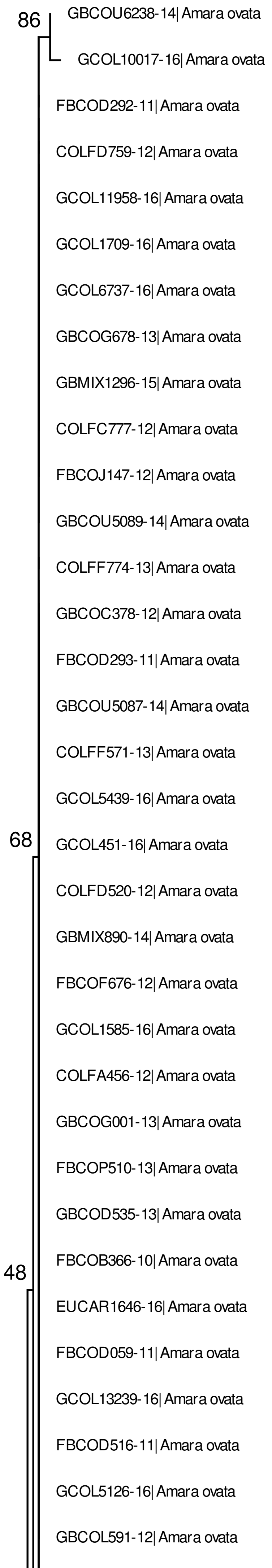
86
GBCOU6238-14| Amara ovata
GCOL10017-16| Amara ovata
FBCOD292-11| Amara ovata
COLFD759-12| Amara ovata
GCOL11958-16| Amara ovata
GCOL1709-16| Amara ovata
GCOL6737-16| Amara ovata
GBCOG678-13| Amara ovata
GBMIX1296-15| Amara ovata
COLFC777-12| Amara ovata
FBCOJ147-12| Amara ovata
GBCOU5089-14| Amara ovata
COLFF774-13| Amara ovata
GBCOC378-12| Amara ovata
FBCOD293-11| Amara ovata
GBCOU5087-14| Amara ovata
COLFF571-13| Amara ovata
GCOL5439-16| Amara ovata
68
GCOL451-16| Amara ovata
COLFD520-12| Amara ovata
GBMIX890-14| Amara ovata
FBCOF676-12| Amara ovata
GCOL1585-16| Amara ovata
COLFA456-12| Amara ovata
GBCOG001-13| Amara ovata
FBCOP510-13| Amara ovata
GBCOD535-13| Amara ovata
48
FBCOB366-10| Amara ovata
EUCAR1646-16| Amara ovata
FBCOD059-11| Amara ovata
GCOL13239-16| Amara ovata
FBCOD516-11| Amara ovata
GCOL5126-16| Amara ovata
GBCOL591-12| Amara ovata

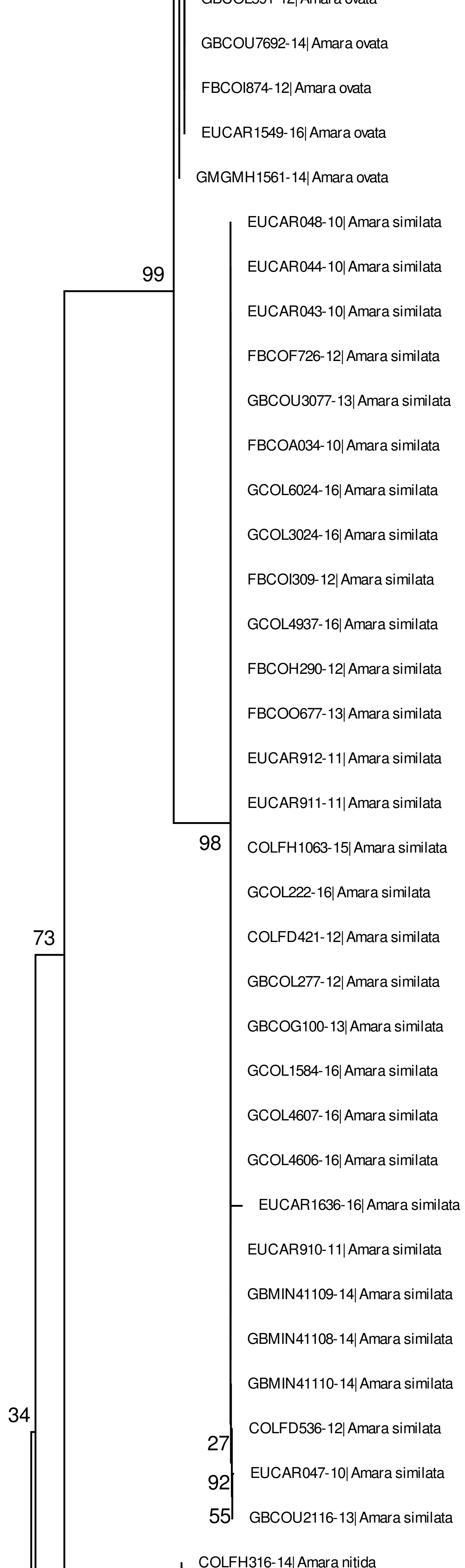
GBCOU7692-14| Amara ovata
FBCOI874-12| Amara ovata
EUCAR1549-16| Amara ovata
GMGMH1561-14| Amara ovata
EUCAR048-10| Amara similata
99
EUCAR044-10| Amara similata
EUCAR043-10| Amara similata
FBCOF726-12| Amara similata
GBCOU3077-13| Amara similata
FBCOA034-10| Amara similata
GCOL6024-16| Amara similata
GCOL3024-16| Amara similata
FBCOI309-12| Amara similata
GCOL4937-16| Amara similata
FBCOH290-12| Amara similata
FBCOO677-13| Amara similata
EUCAR912-11| Amara similata
EUCAR911-11| Amara similata
98
COLFH1063-15| Amara similata
GCOL222-16| Amara similata
73
COLFD421-12| Amara similata
GBCOL277-12| Amara similata
GBCOG100-13| Amara similata
GCOL1584-16| Amara similata
GCOL4607-16| Amara similata
GCOL4606-16| Amara similata
EUCAR1636-16| Amara similata
EUCAR910-11| Amara similata
GBMIN41109-14| Amara similata
GBMIN41108-14| Amara similata
GBMIN41110-14| Amara similata
34
COLFD536-12| Amara similata
27
92
EUCAR047-10| Amara similata
55
GBCOU2116-13| Amara similata
COLFH316-14| Amara nitida

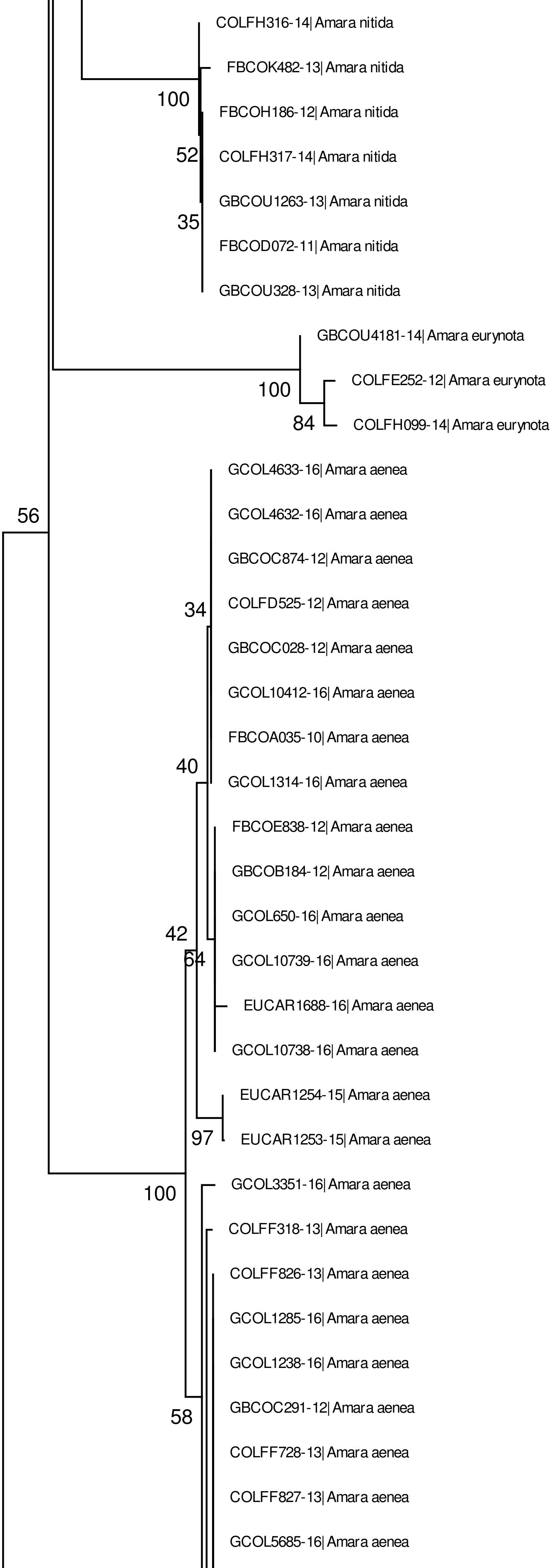

COLFH316-14| Amara nitida
FBCOK482-13| Amara nitida
100
FBCOH186-12| Amara nitida
52
COLFH317-14| Amara nitida
GBCOU1263-13| Amara nitida
35
FBCOD072-11| Amara nitida
GBCOU328-13| Amara nitida
GBCOU4181-14| Amara eurynota
COLFE252-12| Amara eurynota
100
84
COLFH099-14| Amara eurynota
GCOL4633-16| Amara aenea
56
GCOL4632-16| Amara aenea
GBCOC874-12| Amara aenea
34
COLFD525-12| Amara aenea
GBCOC028-12| Amara aenea
GCOL10412-16| Amara aenea
FBCOA035-10| Amara aenea
40
GCOL1314-16| Amara aenea
FBCOE838-12| Amara aenea
GBCOB184-12| Amara aenea
GCOL650-16| Amara aenea
42
64
GCOL10739-16| Amara aenea
EUCAR1688-16| Amara aenea
GCOL10738-16| Amara aenea
EUCAR1254-15| Amara aenea
97
EUCAR1253-15| Amara aenea
GCOL3351-16| Amara aenea
100
COLFF318-13| Amara aenea
COLFF826-13| Amara aenea
GCOL1285-16| Amara aenea
GCOL1238-16| Amara aenea
GBCOC291-12| Amara aenea
58
COLFF728-13| Amara aenea
COLFF827-13| Amara aenea
GCOL5685-16| Amara aenea

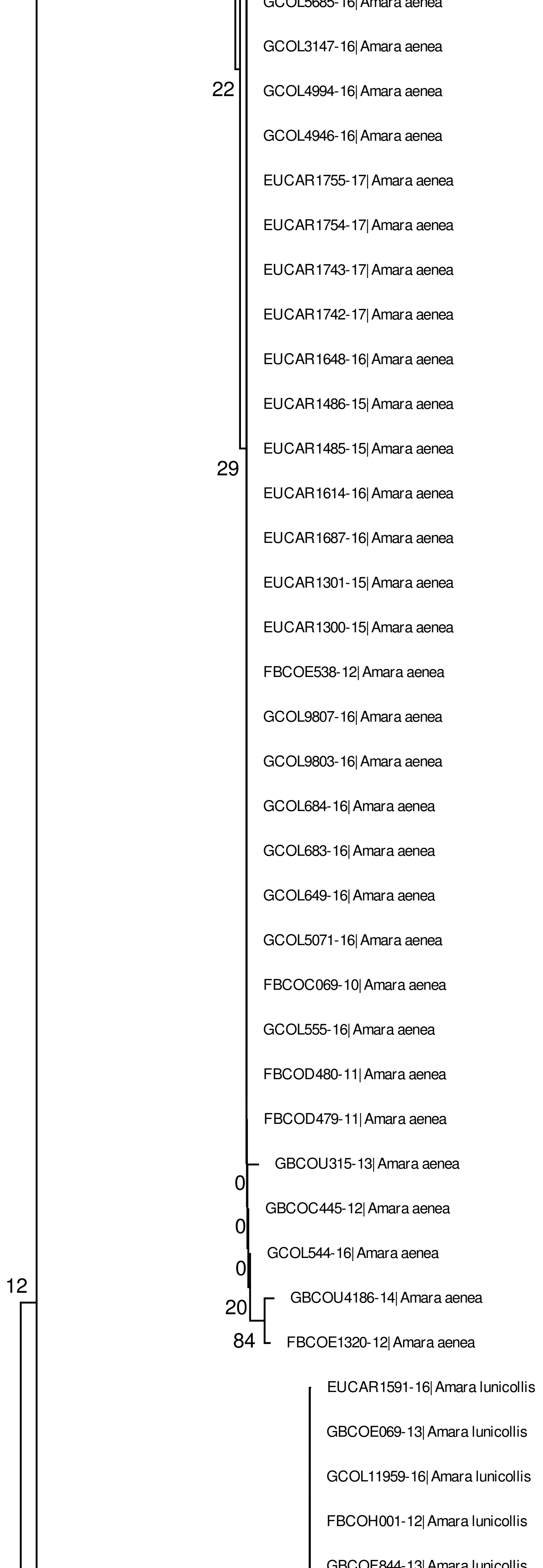
GCOL5685-16| Amara aenea
GCOL3147-16| Amara aenea
22
GCOL4994-16| Amara aenea
GCOL4946-16| Amara aenea
EUCAR1755-17| Amara aenea
EUCAR1754-17| Amara aenea
EUCAR1743-17| Amara aenea
EUCAR1742-17| Amara aenea
EUCAR1648-16| Amara aenea
EUCAR1486-15| Amara aenea
EUCAR1485-15| Amara aenea
29
EUCAR1614-16| Amara aenea
EUCAR1687-16| Amara aenea
EUCAR1301-15| Amara aenea
EUCAR1300-15| Amara aenea
FBCOE538-12| Amara aenea
GCOL9807-16| Amara aenea
GCOL9803-16| Amara aenea
GCOL684-16| Amara aenea
GCOL683-16| Amara aenea
GCOL649-16| Amara aenea
GCOL5071-16| Amara aenea
FBCOC069-10| Amara aenea
GCOL555-16| Amara aenea
FBCOD480-11| Amara aenea
FBCOD479-11| Amara aenea
GBCOU315-13| Amara aenea
0
GBCOC445-12| Amara aenea
0
GCOL544-16| Amara aenea
0
12
GBCOU4186-14| Amara aenea
20
84
FBCOE1320-12| Amara aenea
EUCAR1591-16| Amara lunicollis
GBCOE069-13| Amara lunicollis
GCOL11959-16| Amara lunicollis
FBCOH001-12| Amara lunicollis
GBCOE844-13| Amara lunicollis

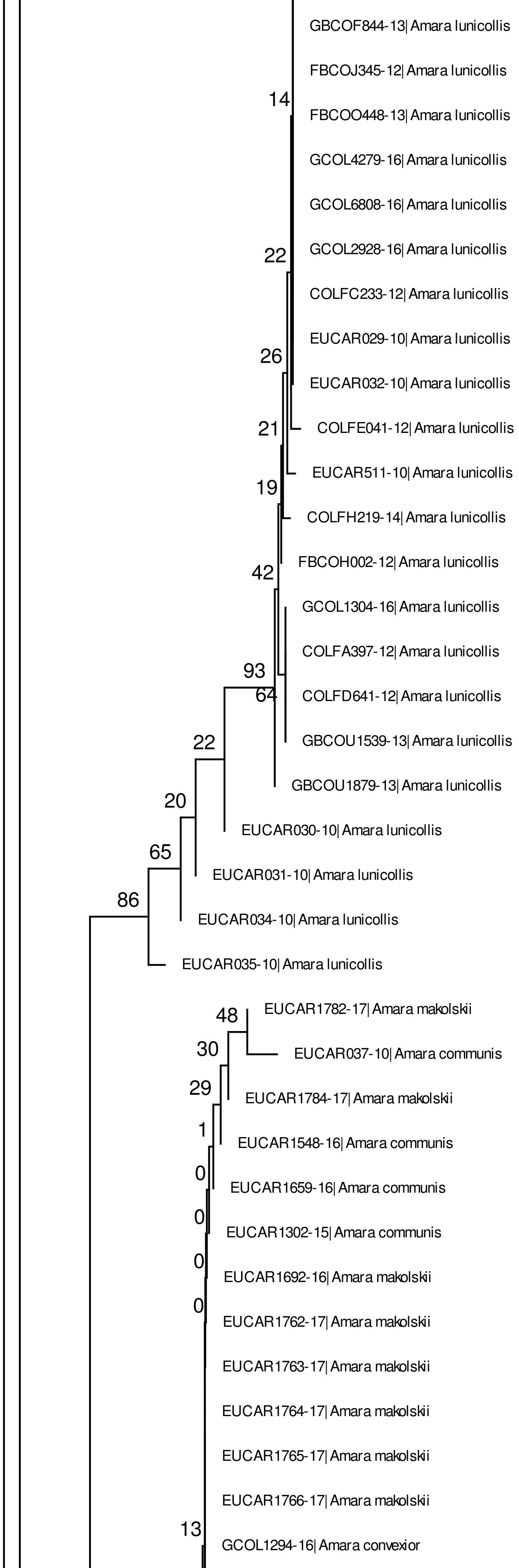

GBCOF844-13| Amara lunicollis
FBCOJ345-12| Amara lunicollis
14
FBCOO448-13| Amara lunicollis
GCOL4279-16| Amara lunicollis
GCOL6808-16| Amara lunicollis
22
GCOL2928-16| Amara lunicollis
COLFC233-12| Amara lunicollis
EUCAR029-10| Amara lunicollis
26
EUCAR032-10| Amara lunicollis
21
COLFE041-12| Amara lunicollis
EUCAR511-10| Amara lunicollis
19
COLFH219-14| Amara lunicollis
FBCOH002-12| Amara lunicollis
42
GCOL1304-16| Amara lunicollis
COLFA397-12| Amara lunicollis
93
64
COLFD641-12| Amara lunicollis
GBCOU1539-13| Amara lunicollis
22
GBCOU1879-13| Amara lunicollis
20
EUCAR030-10| Amara lunicollis
65
EUCAR031-10| Amara lunicollis
86
EUCAR034-10| Amara lunicollis
EUCAR035-10| Amara lunicollis
EUCAR1782-17| Amara makolskii
48
30
EUCAR037-10| Amara communis
29
EUCAR1784-17| Amara makolskii
1
EUCAR1548-16| Amara communis
0
EUCAR1659-16| Amara communis
0
EUCAR1302-15| Amara communis
0
EUCAR1692-16| Amara makolskii
0
EUCAR1762-17| Amara makolskii
EUCAR1763-17| Amara makolskii
EUCAR1764-17| Amara makolskii
EUCAR1765-17| Amara makolskii
EUCAR1766-17| Amara makolskii
13
GCOL1294-16| Amara convexior

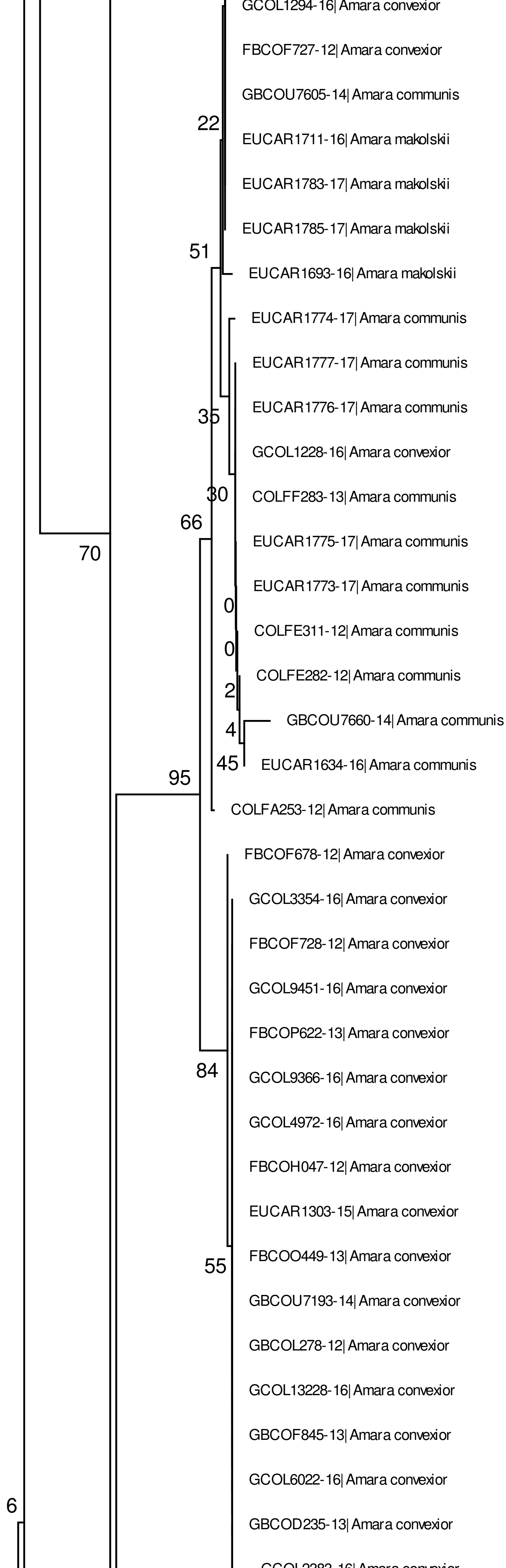
GCOL1294-16| Amara convexior
FBCOF727-12| Amara convexior
GBCOU7605-14| Amara communis
22
EUCAR1711-16| Amara makolskii
EUCAR1783-17| Amara makolskii
EUCAR1785-17| Amara makolskii
51
EUCAR1693-16| Amara makolskii
EUCAR1774-17| Amara communis
EUCAR1777-17| Amara communis
EUCAR1776-17| Amara communis
35
GCOL1228-16| Amara convexior
30
COLFF283-13| Amara communis
66
EUCAR1775-17| Amara communis
70
EUCAR1773-17| Amara communis
0
COLFE311-12| Amara communis
0
COLFE282-12| Amara communis
2
GBCOU7660-14| Amara communis
4
45
EUCAR1634-16| Amara communis
95
COLFA253-12| Amara communis
FBCOF678-12| Amara convexior
GCOL3354-16| Amara convexior
FBCOF728-12| Amara convexior
GCOL9451-16| Amara convexior
FBCOP622-13| Amara convexior
84
GCOL9366-16| Amara convexior
GCOL4972-16| Amara convexior
FBCOH047-12| Amara convexior
EUCAR1303-15| Amara convexior
FBCOO449-13| Amara convexior
55
GBCOU7193-14| Amara convexior
GBCOL278-12| Amara convexior
GCOL13228-16| Amara convexior
GBCOF845-13| Amara convexior
GCOL6022-16| Amara convexior
6
GBCOD235-13| Amara convexior

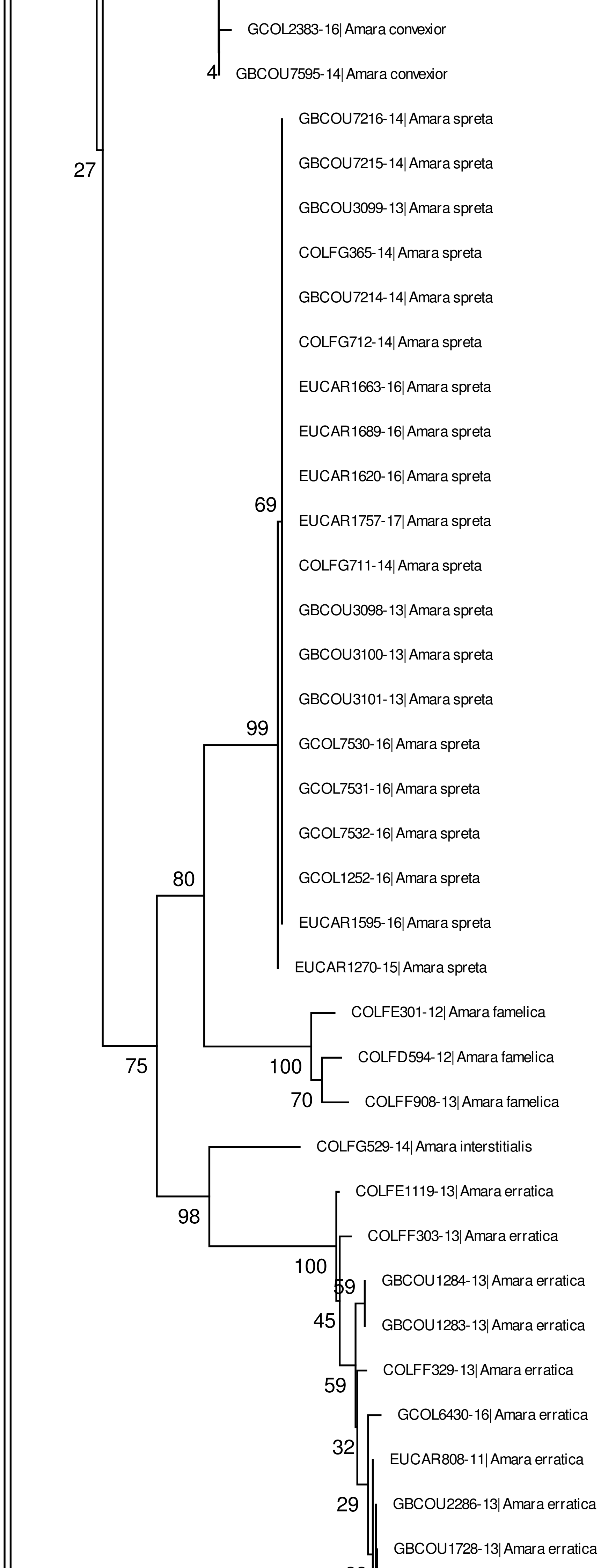
GCOL2383-16| Amara convexior
4
GBCOU7595-14| Amara convexior
GBCOU7216-14| Amara spreta
27
GBCOU7215-14| Amara spreta
GBCOU3099-13| Amara spreta
COLFG365-14| Amara spreta
GBCOU7214-14| Amara spreta
COLFG712-14| Amara spreta
EUCAR1663-16| Amara spreta
EUCAR1689-16| Amara spreta
EUCAR1620-16| Amara spreta
69
EUCAR1757-17| Amara spreta
COLFG711-14| Amara spreta
GBCOU3098-13| Amara spreta
GBCOU3100-13| Amara spreta
GBCOU3101-13| Amara spreta
99
GCOL7530-16| Amara spreta
GCOL7531-16| Amara spreta
GCOL7532-16| Amara spreta
80
GCOL1252-16| Amara spreta
EUCAR1595-16| Amara spreta
EUCAR1270-15| Amara spreta
COLFE301-12| Amara famelica
COLFD594-12| Amara famelica
75
100
70
COLFF908-13| Amara famelica
COLFG529-14| Amara interstitialis
COLFE1119-13| Amara erratica
98
COLFF303-13| Amara erratica
100
59
GBCOU1284-13| Amara erratica
45
GBCOU1283-13| Amara erratica
COLFF329-13| Amara erratica
59
GCOL6430-16| Amara erratica
32
EUCAR808-11| Amara erratica
29
GBCOU2286-13| Amara erratica
GBCOU1728-13| Amara erratica

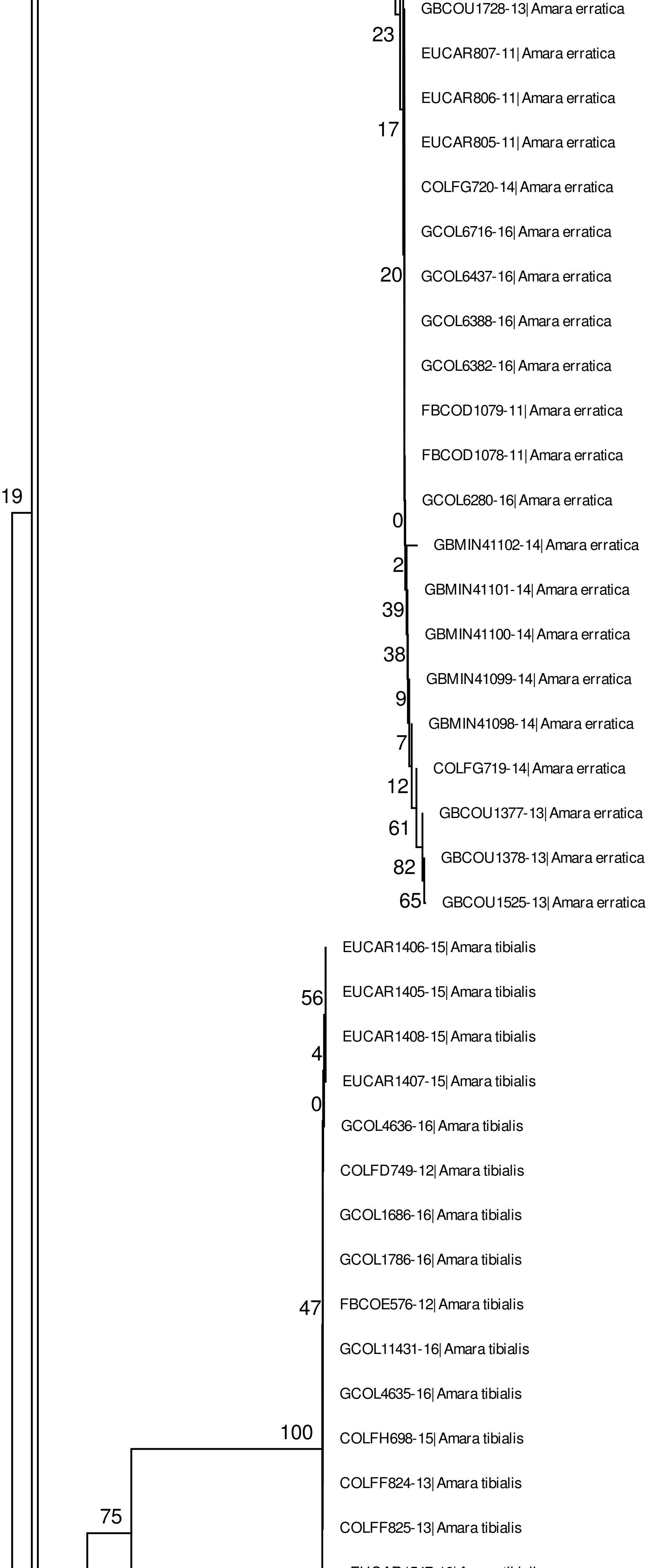
GBCOU1728-13| Amara erratica
23
EUCAR807-11| Amara erratica
EUCAR806-11| Amara erratica
17
EUCAR805-11| Amara erratica
COLFG720-14| Amara erratica
GCOL6716-16| Amara erratica
20
GCOL6437-16| Amara erratica
GCOL6388-16| Amara erratica
GCOL6382-16| Amara erratica
FBCOD1079-11| Amara erratica
FBCOD1078-11| Amara erratica
19
GCOL6280-16| Amara erratica
0
GBMIN41102-14| Amara erratica
2
GBMIN41101-14| Amara erratica
39
GBMIN41100-14| Amara erratica
38
GBMIN41099-14| Amara erratica
9
GBMIN41098-14| Amara erratica
7
COLFG719-14| Amara erratica
12
GBCOU1377-13| Amara erratica
61
GBCOU1378-13| Amara erratica
82
65
GBCOU1525-13| Amara erratica
EUCAR1406-15| Amara tibialis
56
EUCAR1405-15| Amara tibialis
EUCAR1408-15| Amara tibialis
4
EUCAR1407-15| Amara tibialis
0
GCOL4636-16| Amara tibialis
COLFD749-12| Amara tibialis
GCOL1686-16| Amara tibialis
GCOL1786-16| Amara tibialis
47
FBCOE576-12| Amara tibialis
GCOL11431-16| Amara tibialis
GCOL4635-16| Amara tibialis
100
COLFH698-15| Amara tibialis
COLFF824-13| Amara tibialis
75
COLFF825-13| Amara tibialis

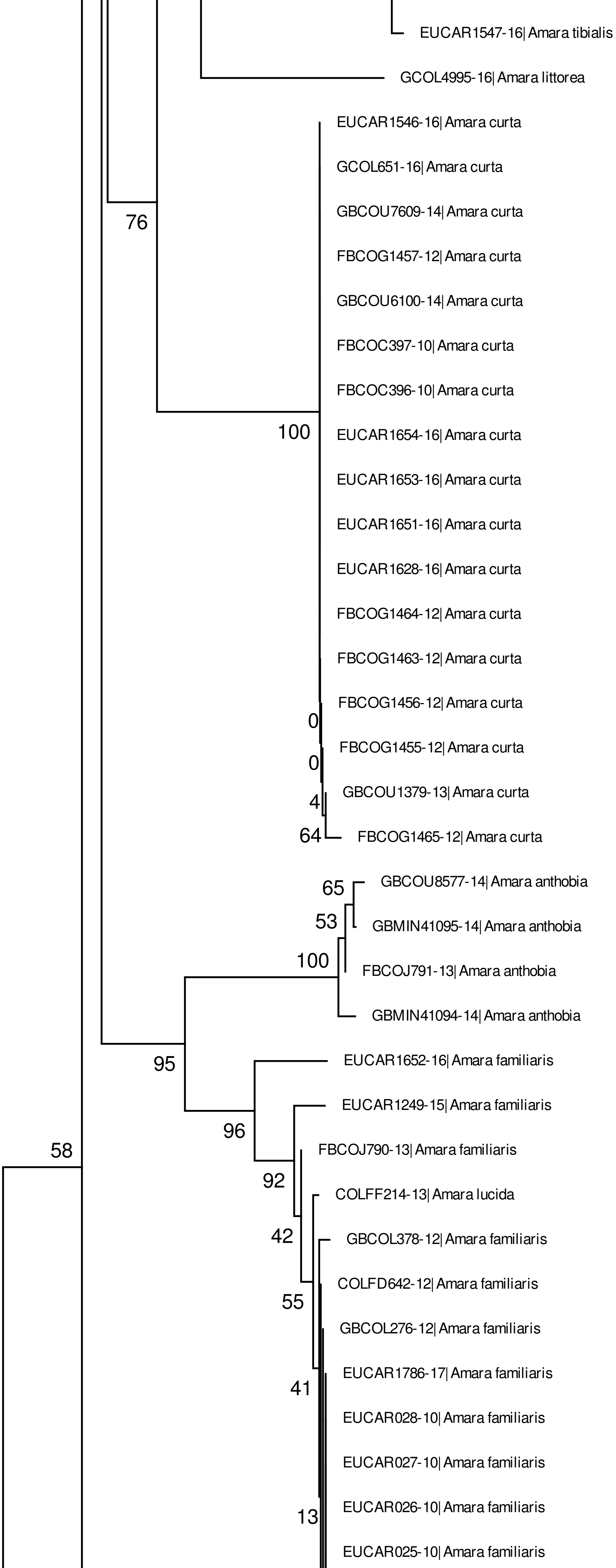
EUCAR1547-16| Amara tibialis
GCOL4995-16| Amara littorea
EUCAR1546-16| Amara curta
GCOL651-16| Amara curta
GBCOU7609-14| Amara curta
FBCOG1457-12| Amara curta
GBCOU6100-14| Amara curta
FBCOC397-10| Amara curta
FBCOC396-10| Amara curta
EUCAR1654-16| Amara curta
EUCAR1653-16| Amara curta
EUCAR1651-16| Amara curta
EUCAR1628-16| Amara curta
FBCOG1464-12| Amara curta
FBCOG1463-12| Amara curta
FBCOG1456-12| Amara curta
FBCOG1455-12| Amara curta
GBCOU1379-13| Amara curta
FBCOG1465-12| Amara curta
GBCOU8577-14| Amara anthobia
GBMIN41095-14| Amara anthobia
FBCOJ791-13| Amara anthobia
GBMIN41094-14| Amara anthobia
EUCAR1652-16| Amara familiaris
EUCAR1249-15| Amara familiaris
FBCOJ790-13| Amara familiaris
COLFF214-13| Amara lucida
GBCOL378-12| Amara familiaris
COLFD642-12| Amara familiaris
GBCOL276-12| Amara familiaris
EUCAR1786-17| Amara familiaris
EUCAR028-10| Amara familiaris
EUCAR027-10| Amara familiaris
EUCAR026-10| Amara familiaris
EUCAR025-10| Amara familiaris
76
100
0
0
4
64
65
53
100
95
96
58
92
42
55
41
13

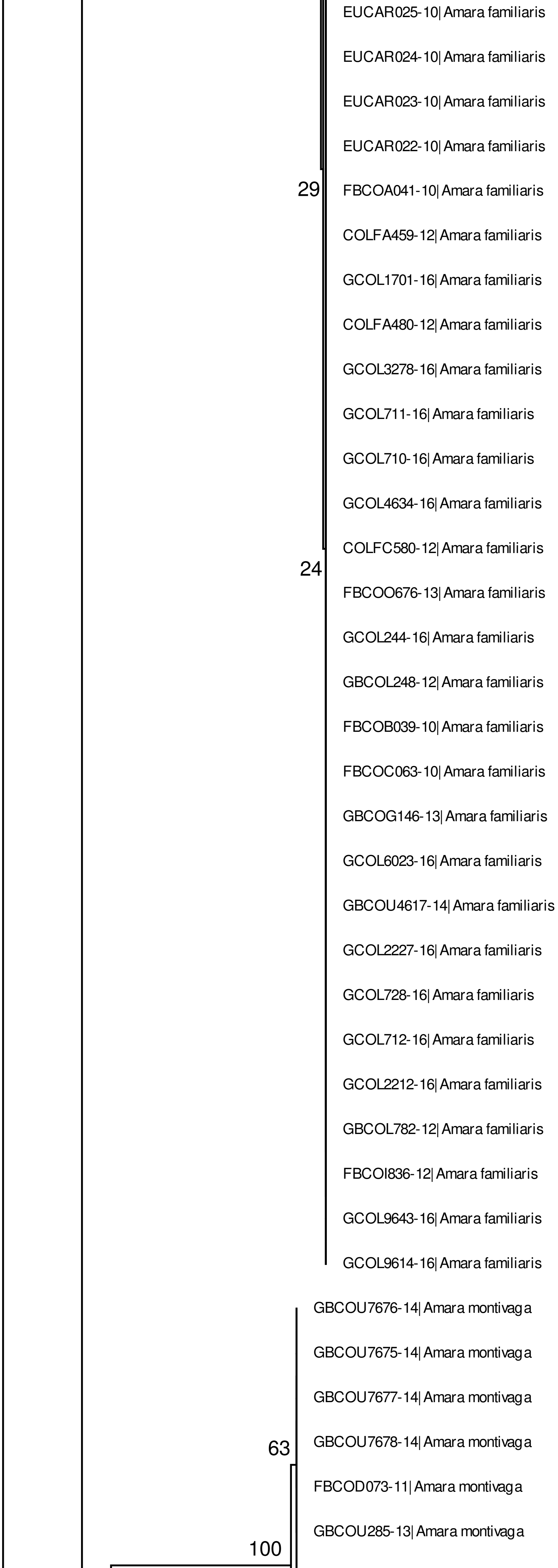

EUCAR025-10| Amara familiaris
EUCAR024-10| Amara familiaris
EUCAR023-10| Amara familiaris
EUCAR022-10| Amara familiaris
29
FBCOA041-10| Amara familiaris
COLFA459-12| Amara familiaris
GCOL1701-16| Amara familiaris
COLFA480-12| Amara familiaris
GCOL3278-16| Amara familiaris
GCOL711-16| Amara familiaris
GCOL710-16| Amara familiaris
GCOL4634-16| Amara familiaris
COLFC580-12| Amara familiaris
24
FBCOO676-13| Amara familiaris
GCOL244-16| Amara familiaris
GBCOL248-12| Amara familiaris
FBCOB039-10| Amara familiaris
FBCOC063-10| Amara familiaris
GBCOG146-13| Amara familiaris
GCOL6023-16| Amara familiaris
GBCOU4617-14| Amara familiaris
GCOL2227-16| Amara familiaris
GCOL728-16| Amara familiaris
GCOL712-16| Amara familiaris
GCOL2212-16| Amara familiaris
GBCOL782-12| Amara familiaris
FBCOI836-12| Amara familiaris
GCOL9643-16| Amara familiaris
GCOL9614-16| Amara familiaris
GBCOU7676-14| Amara montivaga
GBCOU7675-14| Amara montivaga
GBCOU7677-14| Amara montivaga
63
GBCOU7678-14| Amara montivaga
FBCOD073-11| Amara montivaga
GBCOU285-13| Amara montivaga
100

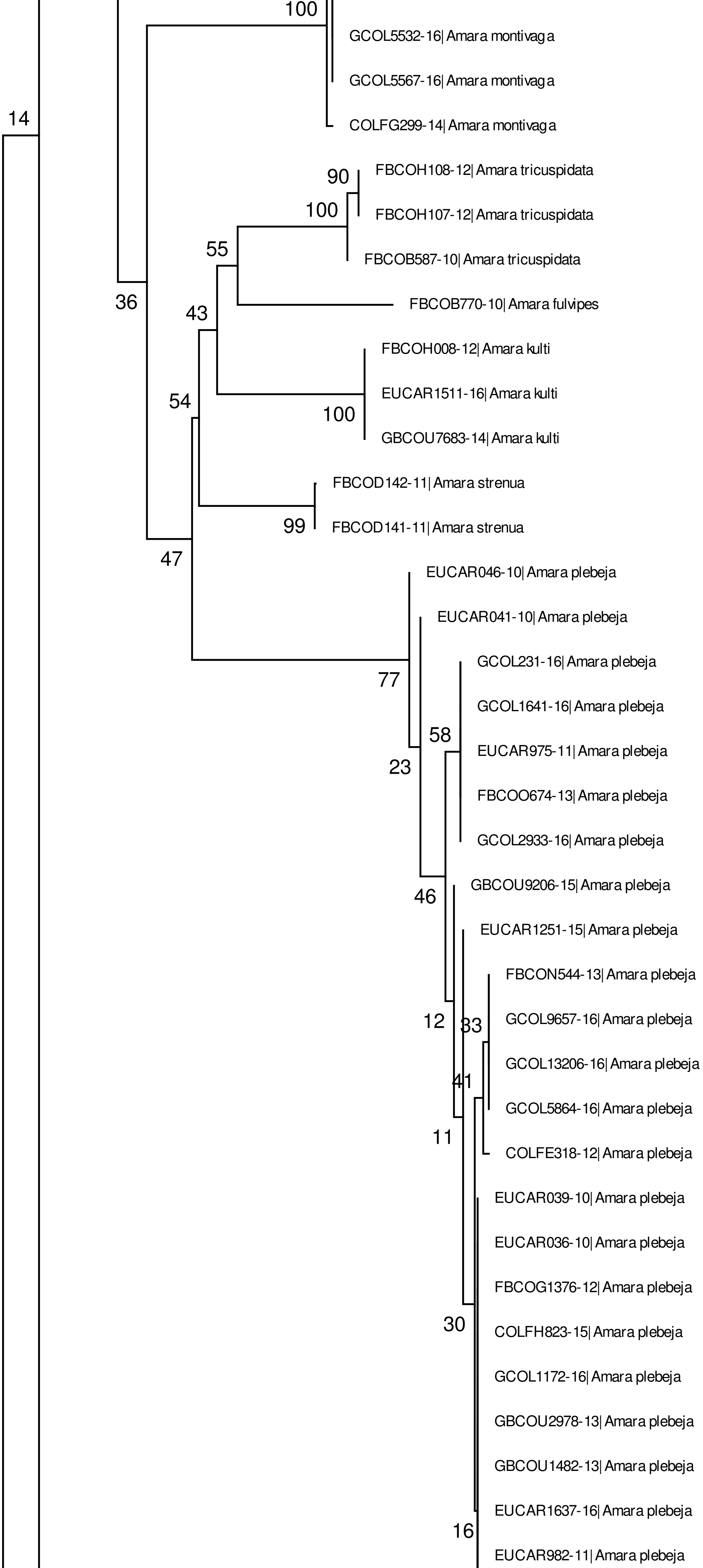

100
GCOL5532-16| Amara montivaga
GCOL5567-16| Amara montivaga
14
COLFG299-14| Amara montivaga
90
FBCOH108-12| Amara tricuspidata
100
FBCOH107-12| Amara tricuspidata
55
FBCOB587-10| Amara tricuspidata
36
43
FBCOB770-10| Amara fulvipes
FBCOH008-12| Amara kulti
54
EUCAR1511-16| Amara kulti
100
GBCOU7683-14| Amara kulti
FBCOD142-11| Amara strenua
99
FBCOD141-11| Amara strenua
47
EUCAR046-10| Amara plebeja
EUCAR041-10| Amara plebeja
GCOL231-16| Amara plebeja
77
GCOL1641-16| Amara plebeja
58
EUCAR975-11| Amara plebeja
23
FBCOO674-13| Amara plebeja
GCOL2933-16| Amara plebeja
GBCOU9206-15| Amara plebeja
46
EUCAR1251-15| Amara plebeja
FBCON544-13| Amara plebeja
12
33
GCOL9657-16| Amara plebeja
GCOL13206-16| Amara plebeja
41
GCOL5864-16| Amara plebeja
11
COLFE318-12| Amara plebeja
EUCAR039-10| Amara plebeja
EUCAR036-10| Amara plebeja
FBCOG1376-12| Amara plebeja
30
COLFH823-15| Amara plebeja
GCOL1172-16| Amara plebeja
GBCOU2978-13| Amara plebeja
GBCOU1482-13| Amara plebeja
EUCAR1637-16| Amara plebeja
16
EUCAR982-11| Amara plebeja

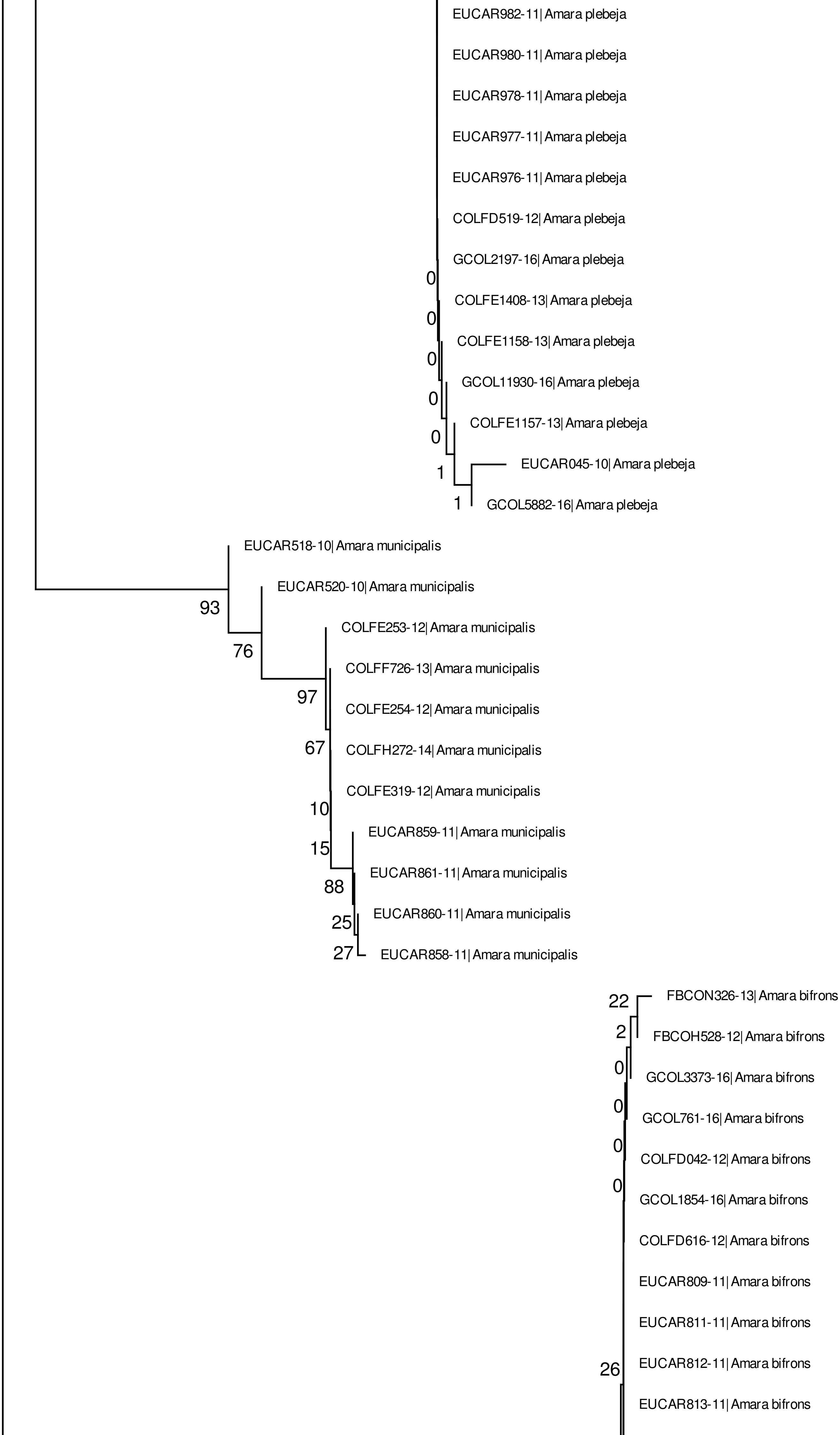

EUCAR982-11| Amara plebeja
EUCAR980-11| Amara plebeja
EUCAR978-11| Amara plebeja
EUCAR977-11| Amara plebeja
EUCAR976-11| Amara plebeja
COLFD519-12| Amara plebeja
GCOL2197-16| Amara plebeja
0
COLFE1408-13| Amara plebeja
0
COLFE1158-13| Amara plebeja
0
GCOL11930-16| Amara plebeja
0
COLFE1157-13| Amara plebeja
0
EUCAR045-10| Amara plebeja
1
GCOL5882-16| Amara plebeja
1
EUCAR518-10| Amara municipalis
EUCAR520-10| Amara municipalis
93
COLFE253-12| Amara municipalis
76
COLFF726-13| Amara municipalis
97
COLFE254-12| Amara municipalis
67
COLFH272-14| Amara municipalis
COLFE319-12| Amara municipalis
10
EUCAR859-11| Amara municipalis
15
EUCAR861-11| Amara municipalis
88
EUCAR860-11| Amara municipalis
25
27
EUCAR858-11| Amara municipalis
22
FBCON326-13| Amara bifrons
2
FBCOH528-12| Amara bifrons
0
GCOL3373-16| Amara bifrons
0
GCOL761-16| Amara bifrons
0
COLFD042-12| Amara bifrons
0
GCOL1854-16| Amara bifrons
COLFD616-12| Amara bifrons
EUCAR809-11| Amara bifrons
EUCAR811-11| Amara bifrons
26
EUCAR812-11| Amara bifrons
EUCAR813-11| Amara bifrons

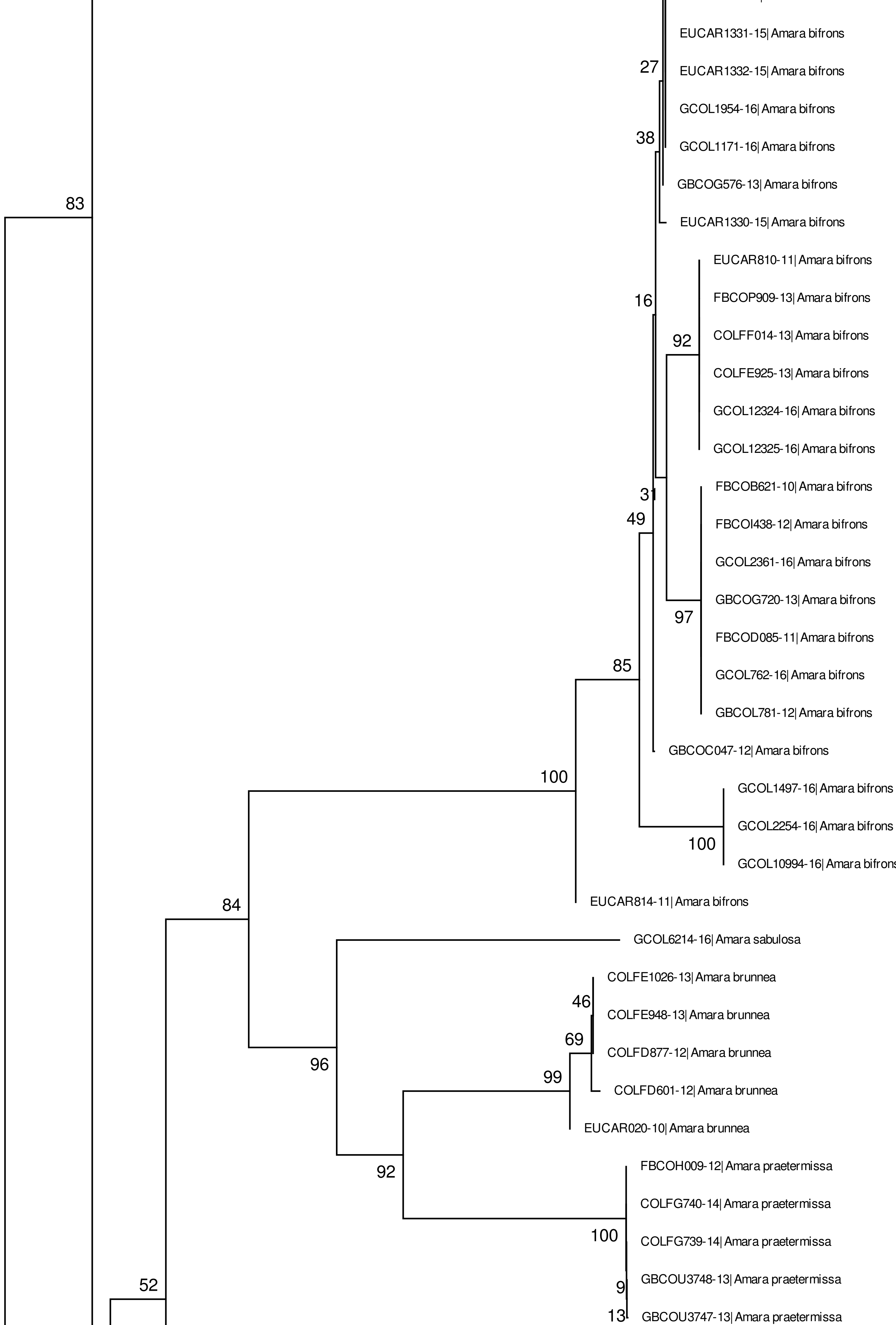

EUCAR1331-15| Amara bifrons
27
EUCAR1332-15| Amara bifrons
GCOL1954-16| Amara bifrons
38
GCOL1171-16| Amara bifrons
83
GBCOG576-13| Amara bifrons
EUCAR1330-15| Amara bifrons
EUCAR810-11| Amara bifrons
16
FBCOP909-13| Amara bifrons
92
COLFF014-13| Amara bifrons
COLFE925-13| Amara bifrons
GCOL12324-16| Amara bifrons
GCOL12325-16| Amara bifrons
FBCOB621-10| Amara bifrons
31
49
FBCOI438-12| Amara bifrons
GCOL2361-16| Amara bifrons
GBCOG720-13| Amara bifrons
97
FBCOD085-11| Amara bifrons
85
GCOL762-16| Amara bifrons
GBCOL781-12| Amara bifrons
GBCOC047-12| Amara bifrons
100
GCOL1497-16| Amara bifrons
GCOL2254-16| Amara bifrons
100
GCOL10994-16| Amara bifrons
84
EUCAR814-11| Amara bifrons
GCOL6214-16| Amara sabulosa
COLFE1026-13| Amara brunnea
46
COLFE948-13| Amara brunnea
69
COLFD877-12| Amara brunnea
96
99
COLFD601-12| Amara brunnea
EUCAR020-10| Amara brunnea
92
FBCOH009-12| Amara praetermissa
COLFG740-14| Amara praetermissa
100
COLFG739-14| Amara praetermissa
52
9
GBCOU3748-13| Amara praetermissa
13
GBCOU3747-13| Amara praetermissa

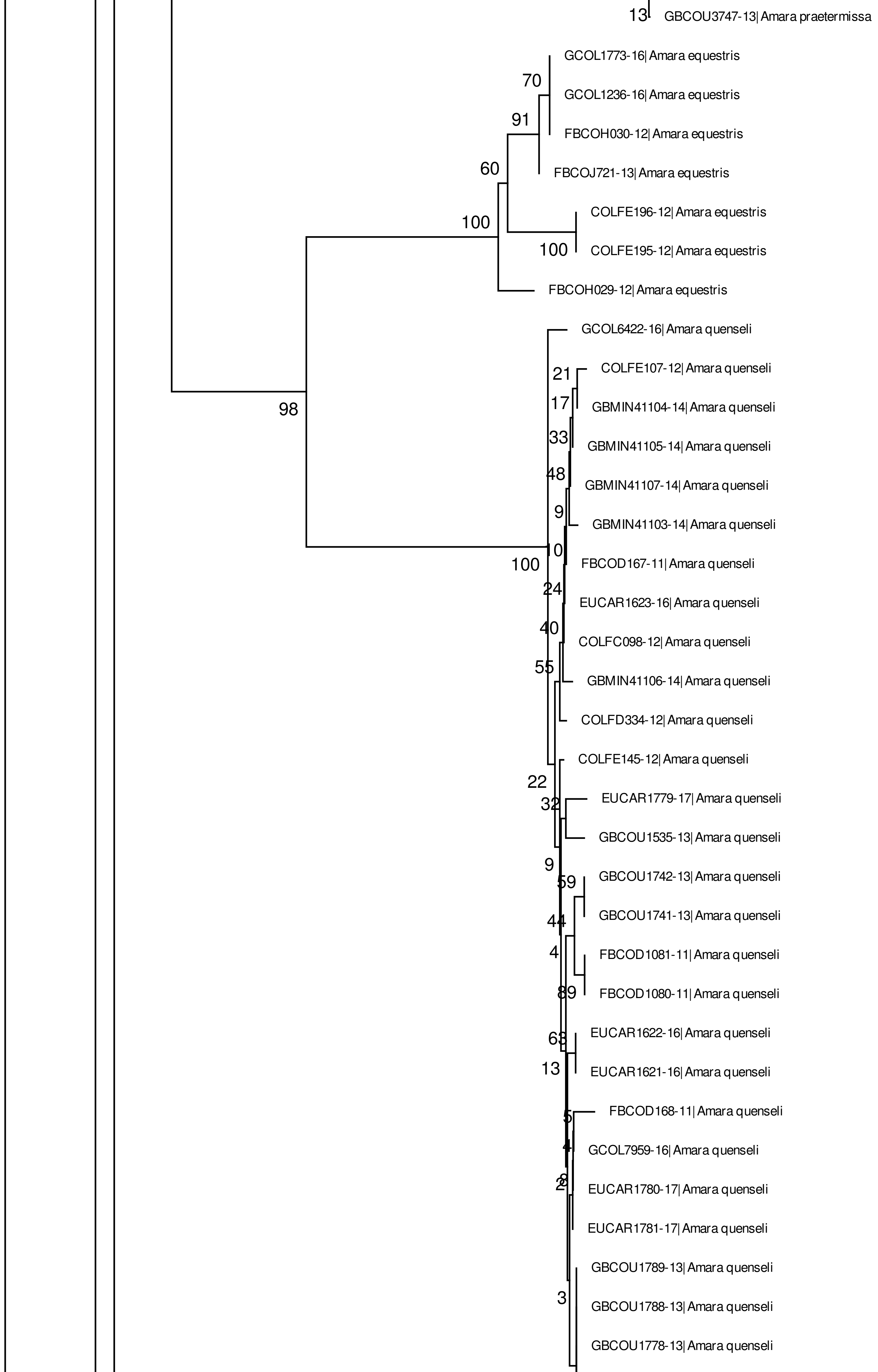
13
GBCOU3747-13| Amara praetermissa
GCOL1773-16| Amara equestris
70
GCOL1236-16| Amara equestris
91
FBCOH030-12| Amara equestris
60
FBCOJ721-13| Amara equestris
COLFE196-12| Amara equestris
100
100
COLFE195-12| Amara equestris
FBCOH029-12| Amara equestris
GCOL6422-16| Amara quenseli
21
COLFE107-12| Amara quenseli
17
GBMIN41104-14| Amara quenseli
98
33
GBMIN41105-14| Amara quenseli
48
GBMIN41107-14| Amara quenseli
9
GBMIN41103-14| Amara quenseli
10
100
FBCOD167-11| Amara quenseli
24
EUCAR1623-16| Amara quenseli
40
COLFC098-12| Amara quenseli
55
GBMIN41106-14| Amara quenseli
COLFD334-12| Amara quenseli
COLFE145-12| Amara quenseli
22
32
EUCAR1779-17| Amara quenseli
GBCOU1535-13| Amara quenseli
9
59
GBCOU1742-13| Amara quenseli
44
GBCOU1741-13| Amara quenseli
4
FBCOD1081-11| Amara quenseli
89
FBCOD1080-11| Amara quenseli
63
EUCAR1622-16| Amara quenseli
13
EUCAR1621-16| Amara quenseli
5
FBCOD168-11| Amara quenseli
4
GCOL7959-16| Amara quenseli
2
8
EUCAR1780-17| Amara quenseli
EUCAR1781-17| Amara quenseli
GBCOU1789-13| Amara quenseli
3
GBCOU1788-13| Amara quenseli
GBCOU1778-13| Amara quenseli

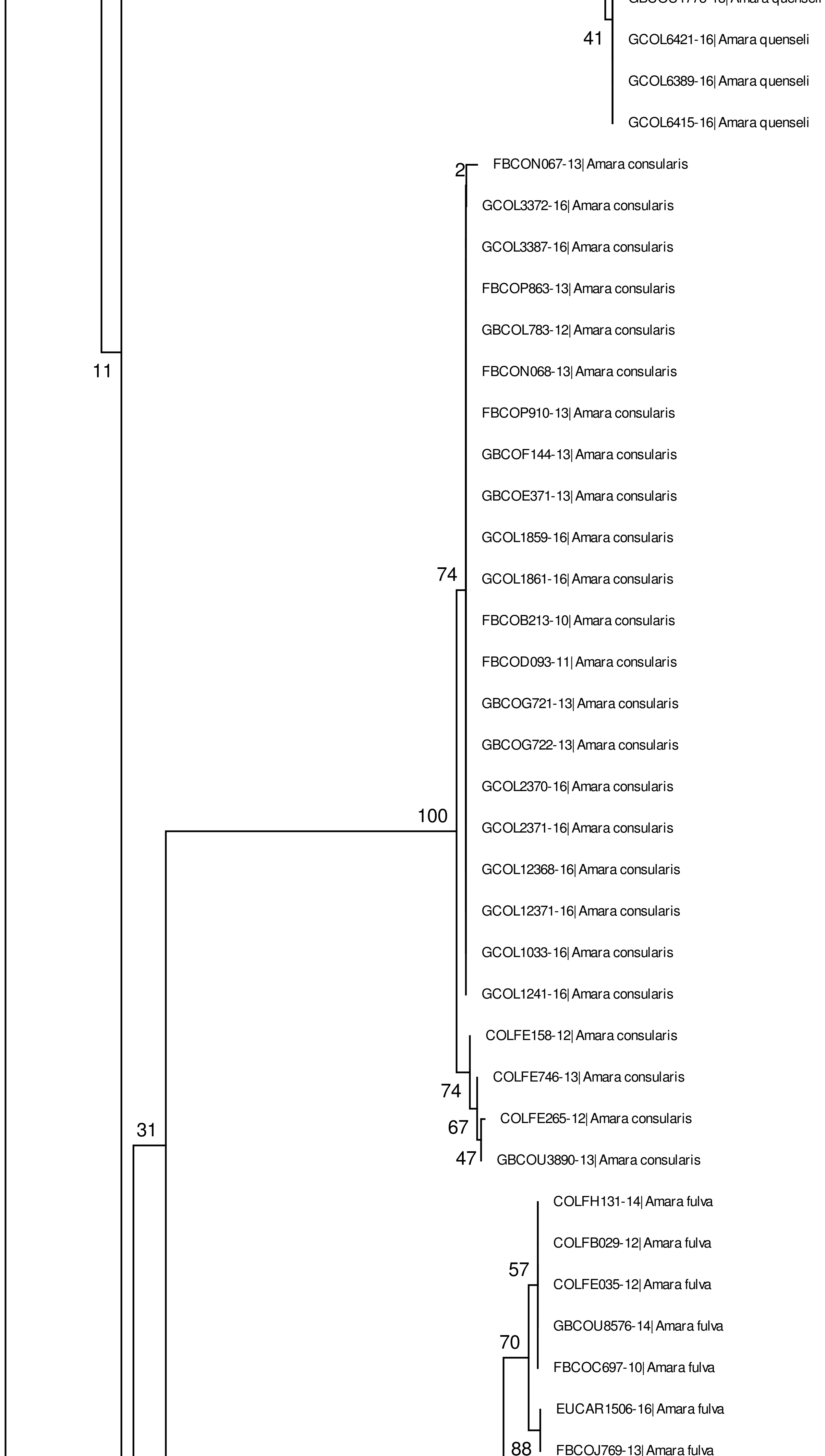
41
GCOL6421-16| Amara quenseli
GCOL6389-16| Amara quenseli
GCOL6415-16| Amara quenseli
2
FBCON067-13| Amara consularis
GCOL3372-16| Amara consularis
GCOL3387-16| Amara consularis
FBCOP863-13| Amara consularis
GBCOL783-12| Amara consularis
11
FBCON068-13| Amara consularis
FBCOP910-13| Amara consularis
GBCOF144-13| Amara consularis
GBCOE371-13| Amara consularis
GCOL1859-16| Amara consularis
74
GCOL1861-16| Amara consularis
FBCOB213-10| Amara consularis
FBCOD093-11| Amara consularis
GBCOG721-13| Amara consularis
GBCOG722-13| Amara consularis
GCOL2370-16| Amara consularis
100
GCOL2371-16| Amara consularis
GCOL12368-16| Amara consularis
GCOL12371-16| Amara consularis
GCOL1033-16| Amara consularis
GCOL1241-16| Amara consularis
COLFE158-12| Amara consularis
74
COLFE746-13| Amara consularis
31
67
COLFE265-12| Amara consularis
47
GBCOU3890-13| Amara consularis
COLFH131-14| Amara fulva
COLFB029-12| Amara fulva
57
COLFE035-12| Amara fulva
GBCOU8576-14| Amara fulva
70
FBCOC697-10| Amara fulva
EUCAR1506-16| Amara fulva
88
FBCOJ769-13| Amara fulva

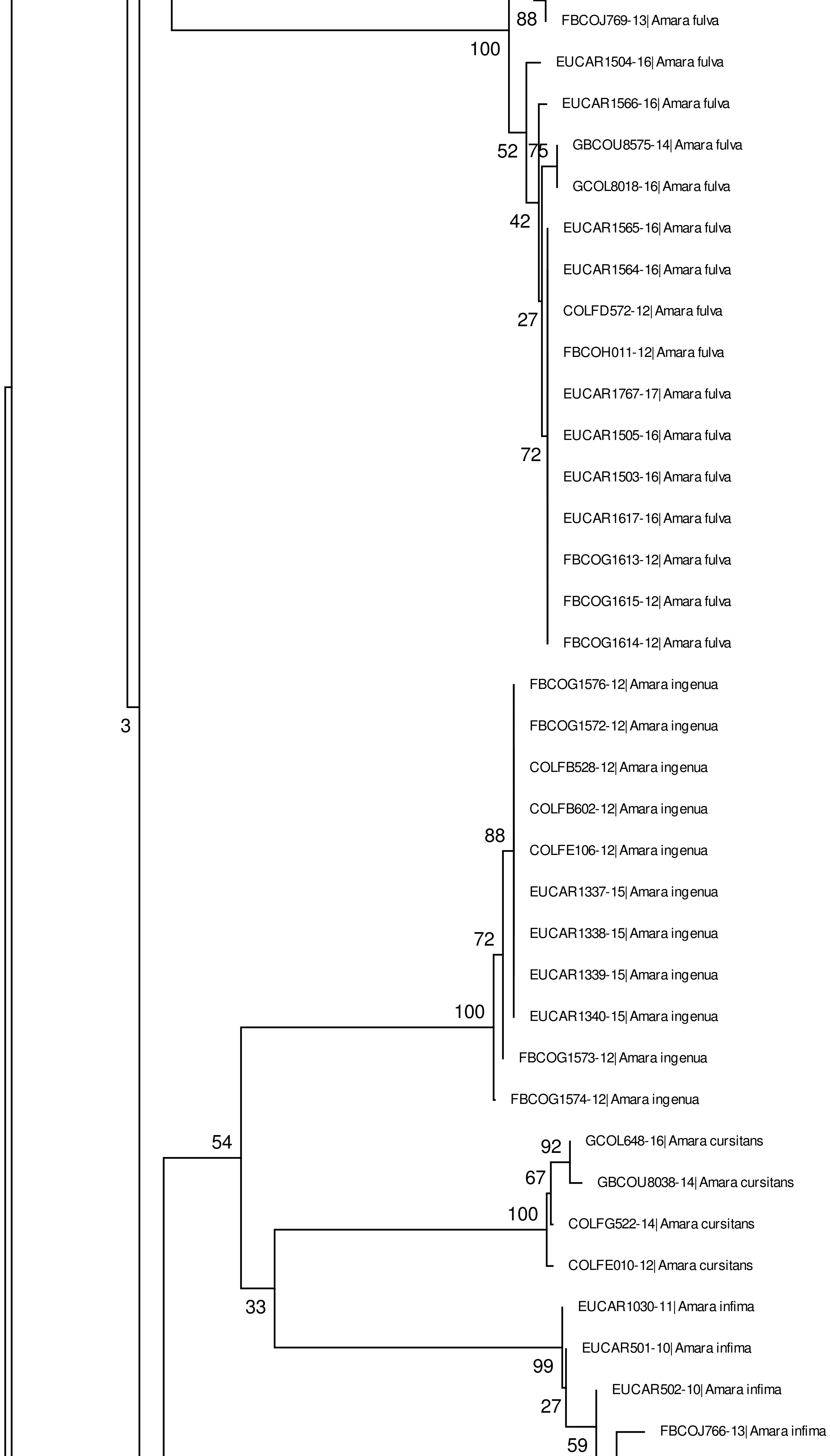

88
FBCOJ769-13| Amara fulva
100
EUCAR1504-16| Amara fulva
EUCAR1566-16| Amara fulva
52
75
GBCOU8575-14| Amara fulva
GCOL8018-16| Amara fulva
42
EUCAR1565-16| Amara fulva
EUCAR1564-16| Amara fulva
27
COLFD572-12| Amara fulva
FBCOH011-12| Amara fulva
EUCAR1767-17| Amara fulva
EUCAR1505-16| Amara fulva
72
EUCAR1503-16| Amara fulva
EUCAR1617-16| Amara fulva
FBCOG1613-12| Amara fulva
FBCOG1615-12| Amara fulva
FBCOG1614-12| Amara fulva
FBCOG1576-12| Amara ingenua
3
FBCOG1572-12| Amara ingenua
COLFB528-12| Amara ingenua
COLFB602-12| Amara ingenua
88
COLFE106-12| Amara ingenua
EUCAR1337-15| Amara ingenua
72
EUCAR1338-15| Amara ingenua
EUCAR1339-15| Amara ingenua
100
EUCAR1340-15| Amara ingenua
FBCOG1573-12| Amara ingenua
FBCOG1574-12| Amara ingenua
54
92
GCOL648-16| Amara cursitans
67
GBCOU8038-14| Amara cursitans
100
COLFG522-14| Amara cursitans
COLFE010-12| Amara cursitans
33
EUCAR1030-11| Amara infima
EUCAR501-10| Amara infima
99
EUCAR502-10| Amara infima
27
FBCOJ766-13| Amara infima
59

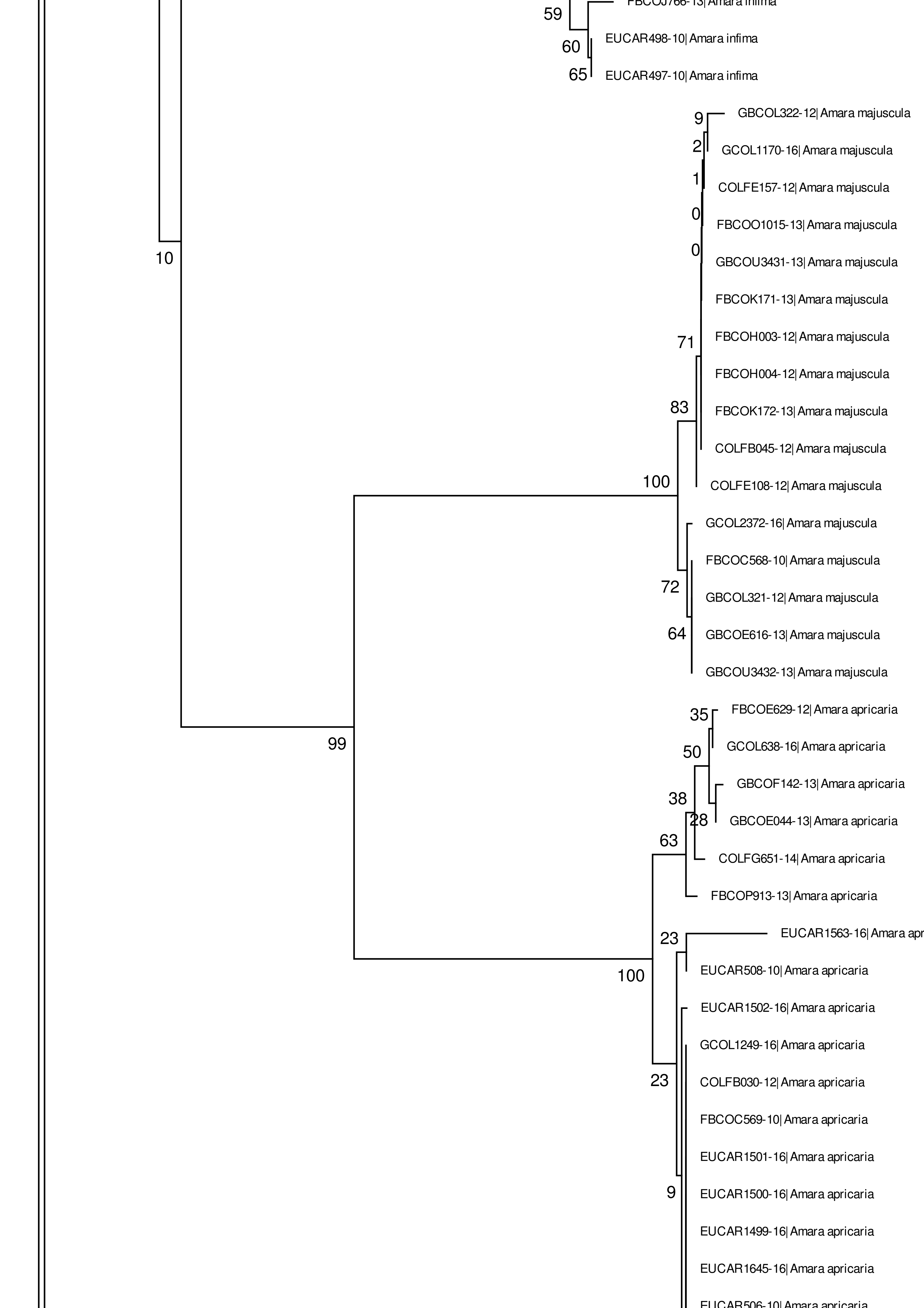
FBCOJ766-13| Amara infima
EUCAR498-10| Amara infima
EUCAR497-10| Amara infima
GBCOL322-12| Amara majuscula
GCOL1170-16| Amara majuscula
COLFE157-12| Amara majuscula
FBCOO1015-13| Amara majuscula
GBCOU3431-13| Amara majuscula
FBCOK171-13| Amara majuscula
FBCOH003-12| Amara majuscula
FBCOH004-12| Amara majuscula
FBCOK172-13| Amara majuscula
COLFB045-12| Amara majuscula
COLFE108-12| Amara majuscula
GCOL2372-16| Amara majuscula
FBCOC568-10| Amara majuscula
GBCOL321-12| Amara majuscula
GBCOE616-13| Amara majuscula
GBCOU3432-13| Amara majuscula
FBCOE629-12| Amara apricaria
GCOL638-16| Amara apricaria
GBCOF142-13| Amara apricaria
GBCOE044-13| Amara apricaria
COLFG651-14| Amara apricaria
FBCOP913-13| Amara apricaria
EUCAR1563-16| Amara apr
EUCAR508-10| Amara apricaria
EUCAR1502-16| Amara apricaria
GCOL1249-16| Amara apricaria
COLFB030-12| Amara apricaria
FBCOC569-10| Amara apricaria
EUCAR1501-16| Amara apricaria
EUCAR1500-16| Amara apricaria
EUCAR1499-16| Amara apricaria
EUCAR1645-16| Amara apricaria
EUCAR506-10| Amara apricaria

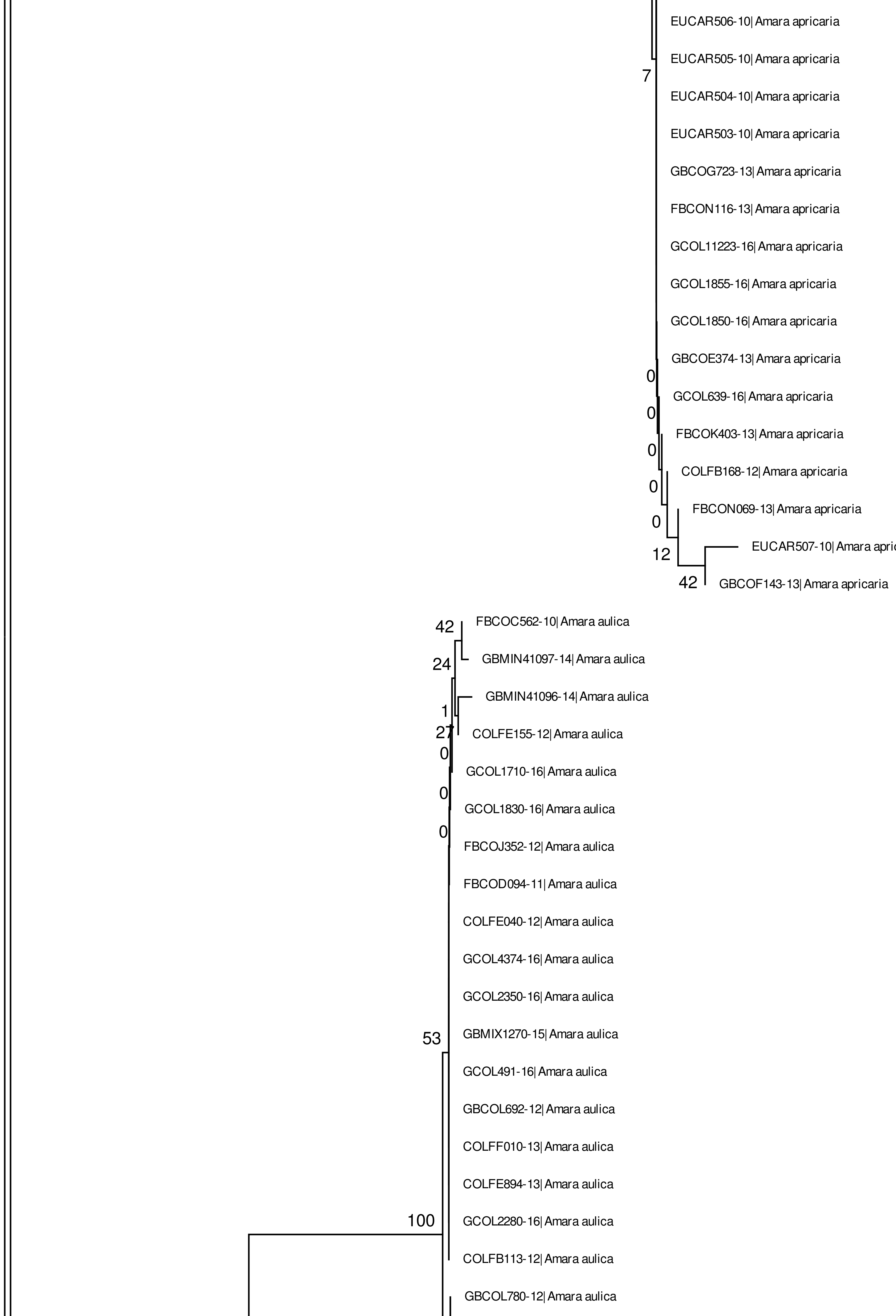

EUCAR506-10| Amara apricaria
EUCAR505-10| Amara apricaria
7
EUCAR504-10| Amara apricaria
EUCAR503-10| Amara apricaria
GBCOG723-13| Amara apricaria
FBCON116-13| Amara apricaria
GCOL11223-16| Amara apricaria
GCOL1855-16| Amara apricaria
GCOL1850-16| Amara apricaria
GBCOE374-13| Amara apricaria
0
GCOL639-16| Amara apricaria
0
FBCOK403-13| Amara apricaria
0
COLFB168-12| Amara apricaria
0
FBCON069-13| Amara apricaria
0
EUCAR507-10| Amara apric
12
42
GBCOF143-13| Amara apricaria
42
FBCOC562-10| Amara aulica
24
GBMIN41097-14| Amara aulica
GBMIN41096-14| Amara aulica
1
27
COLFE155-12| Amara aulica
0
GCOL1710-16| Amara aulica
0
GCOL1830-16| Amara aulica
0
FBCOJ352-12| Amara aulica
FBCOD094-11| Amara aulica
COLFE040-12| Amara aulica
GCOL4374-16| Amara aulica
GCOL2350-16| Amara aulica
53
GBMIX1270-15| Amara aulica
GCOL491-16| Amara aulica
GBCOL692-12| Amara aulica
COLFF010-13| Amara aulica
COLFE894-13| Amara aulica
100
GCOL2280-16| Amara aulica
COLFB113-12| Amara aulica
GBCOL780-12| Amara aulica

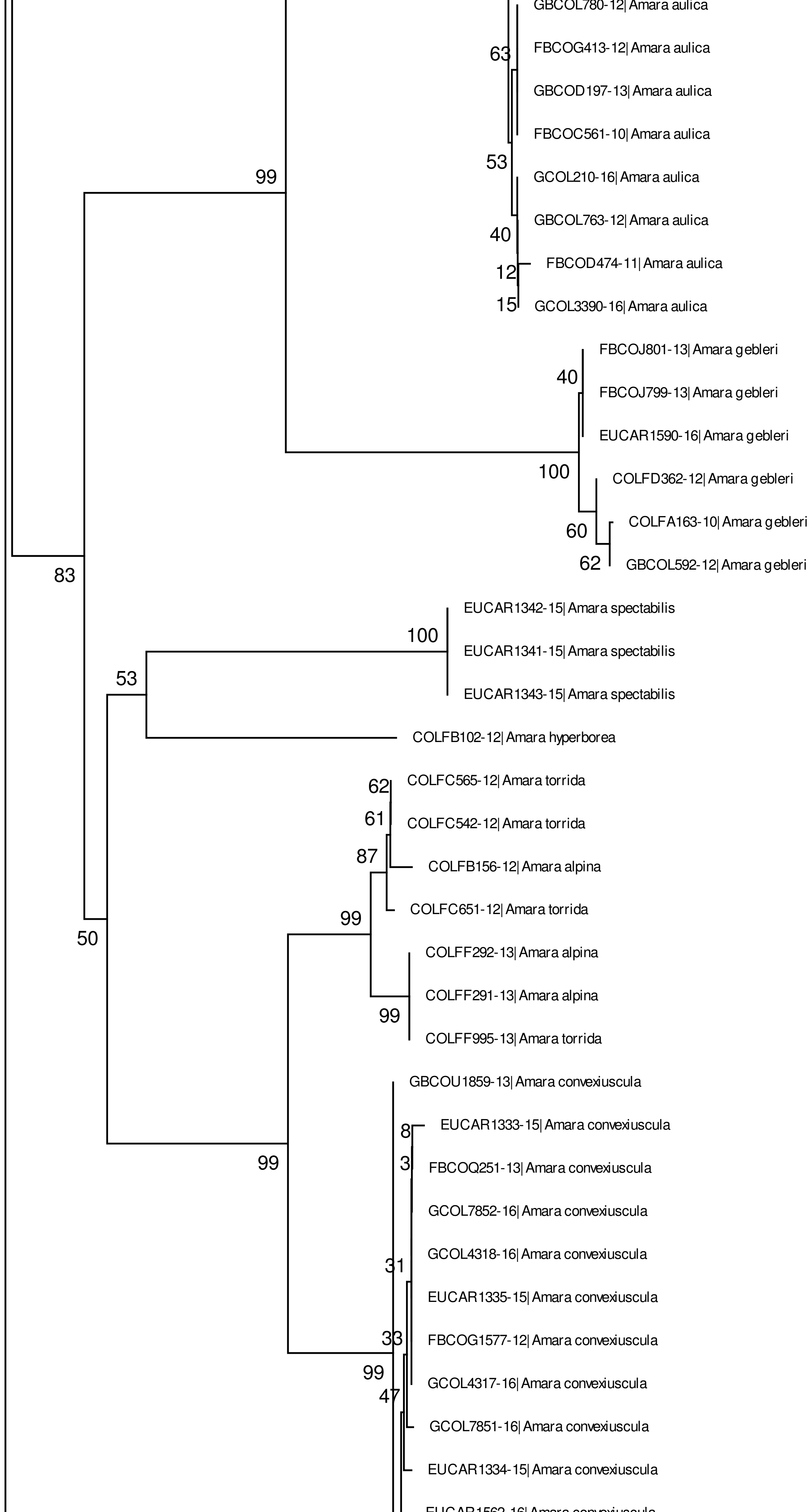
GBCOL780-12| Amara aulica
FBCOG413-12| Amara aulica
GBCOD197-13| Amara aulica
FBCOC561-10| Amara aulica
GCOL210-16| Amara aulica
GBCOL763-12| Amara aulica
FBCOD474-11| Amara aulica
GCOL3390-16| Amara aulica
FBCOJ801-13| Amara gebleri
FBCOJ799-13| Amara gebleri
EUCAR1590-16| Amara gebleri
COLFD362-12| Amara gebleri
COLFA163-10| Amara gebleri
GBCOL592-12| Amara gebleri
EUCAR1342-15| Amara spectabilis
EUCAR1341-15| Amara spectabilis
EUCAR1343-15| Amara spectabilis
COLFB102-12| Amara hyperborea
COLFC565-12| Amara torrida
COLFC542-12| Amara torrida
COLFB156-12| Amara alpina
COLFC651-12| Amara torrida
COLFF292-13| Amara alpina
COLFF291-13| Amara alpina
COLFF995-13| Amara torrida
GBCOU1859-13| Amara convexiuscula
EUCAR1333-15| Amara convexiuscula
FBCOQ251-13| Amara convexiuscula
GCOL7852-16| Amara convexiuscula
GCOL4318-16| Amara convexiuscula
EUCAR1335-15| Amara convexiuscula
FBCOG1577-12| Amara convexiuscula
GCOL4317-16| Amara convexiuscula
GCOL7851-16| Amara convexiuscula
EUCAR1334-15| Amara convexiuscula
63
53
40
12
15
99
40
100
60
62
83
100
53
62
61
87
99
99
50
8
3
31
33
99
47
99

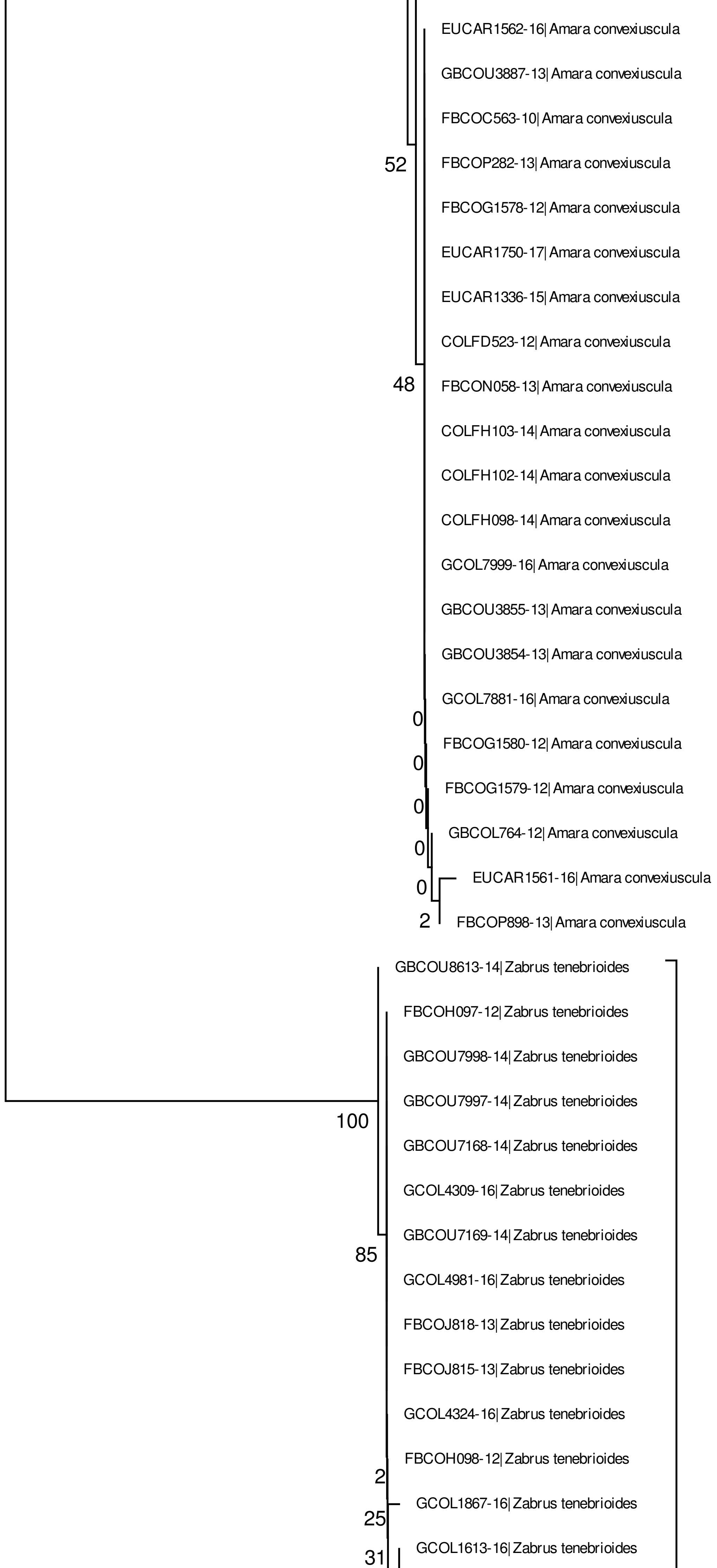
EUCAR1562-16| Amara convexiuscula
GBCOU3887-13| Amara convexiuscula
FBCOC563-10| Amara convexiuscula
52
FBCOP282-13| Amara convexiuscula
FBCOG1578-12| Amara convexiuscula
EUCAR1750-17| Amara convexiuscula
EUCAR1336-15| Amara convexiuscula
COLFD523-12| Amara convexiuscula
48
FBCON058-13| Amara convexiuscula
COLFH103-14| Amara convexiuscula
COLFH102-14| Amara convexiuscula
COLFH098-14| Amara convexiuscula
GCOL7999-16| Amara convexiuscula
GBCOU3855-13| Amara convexiuscula
GBCOU3854-13| Amara convexiuscula
GCOL7881-16| Amara convexiuscula
0
FBCOG1580-12| Amara convexiuscula
0
FBCOG1579-12| Amara convexiuscula
0
GBCOL764-12| Amara convexiuscula
0
EUCAR1561-16| Amara convexiuscula
0
2
FBCOP898-13| Amara convexiuscula
GBCOU8613-14| Zabrus tenebrioides
FBCOH097-12| Zabrus tenebrioides
GBCOU7998-14| Zabrus tenebrioides
GBCOU7997-14| Zabrus tenebrioides
100
GBCOU7168-14| Zabrus tenebrioides
GCOL4309-16| Zabrus tenebrioides
GBCOU7169-14| Zabrus tenebrioides
85
GCOL4981-16| Zabrus tenebrioides
FBCOJ818-13| Zabrus tenebrioides
FBCOJ815-13| Zabrus tenebrioides
GCOL4324-16| Zabrus tenebrioides
FBCOH098-12| Zabrus tenebrioides
2
GCOL1867-16| Zabrus tenebrioides
25
GCOL1613-16| Zabrus tenebrioides
31

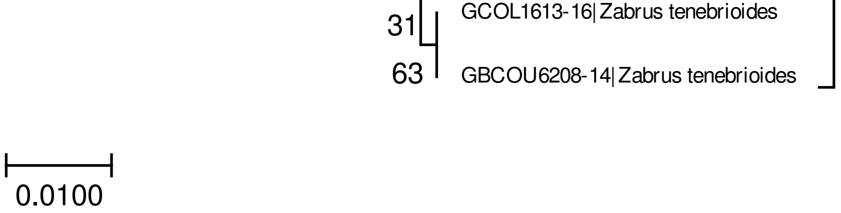
31
GCOL1613-16|Zabrus tenebrioides
63
GBCOU6208-14|Zabrus tenebrioides
0.0100
